# The prominent pervasive oncogenic role and tissue specific permissiveness of RAS gene mutations

**DOI:** 10.1038/s41598-024-76591-8

**Published:** 2024-10-26

**Authors:** Ming Yi, Daniel Soppet, Frank McCormick, Dwight V. Nissley

**Affiliations:** 1 NCI RAS Initiative, Cancer Research Technology Program, Frederick National Laboratory for Cancer Research, Frederick, MD USA; 2 UCSF Helen Diller Family Comprehensive Cancer Center, San Francisco, CA USA

**Keywords:** Cancer, Computational biology and bioinformatics, Genetics

## Abstract

In cancer research, RAS biology has been focused on only a handful of tumor types. While RAS genes have long been suspected as common contributors to a wide spectrum of cancer types, robust evidence is required to firmly establish their critical oncogenic significance. We present a data mining study using DepMap genome-wide CRISPR screening data, which provide substantial evidence to support the prominent pervasive oncogenic role and tissue-specific permissiveness of RAS gene mutations. Differential analysis of CRISPR effect scores identifies K- or N-RAS genes as the most differential gene in contrasts of (K-, N-, combined) RAS mutant versus wild-type cell lines across multiple tissue types. The distinguished tissue-specific pattern of KRAS vs. NRAS as top differential genes in subsets of tissue types and evidence from genome data supported the idea of KRAS- and NRAS-engaged tissue types. To our knowledge, this is the first report of prominent pervasive oncogenic role of RAS mutations revealed by gene dependency data that is beyond the current understanding of the oncogenic role of RAS genes and their well-known involved tissue types. Our findings strongly support RAS mutations as primary oncogenic drivers beyond traditionally recognized cancer types and offer insights into their tissue-specific permissiveness.

**Supplementary Information:**

The online version contains supplementary material available at 10.1038/s41598-024-76591-8.

## Introduction

The RAS genes (KRAS, NRAS, HRAS) hold significant historical importance as they were the first human oncogenes discovered in the field of cancer research. Moreover, they stand out as the most frequently mutated oncogenes in human cancers^1–4^. Notably, KRAS exhibits the highest frequency of mutations in Pancreatic Adenocarcinoma (PAAD), followed by Colon Adenocarcinoma (COAD) and Lung Adenocarcinoma (LUAD), which are also tumor types that garner considerable attention from the cancer research community^1,4^.

However, it is crucial to recognize that RAS gene mutations occur in numerous other cancer types and are believed to play a pivotal role in the oncogenesis of approximately 20% of human cancers^1,2,4^. This suggests that RAS gene mutations may have a more extensive impact as oncogenic drivers than our current research focus suggests. Despite our ongoing efforts to advance our understanding of RAS biology, with a better understanding of RAS-engaged cancer types^2,5,6^, computational studies have also revealed that RAS genes possess potential oncogenic driver properties in various tumor or tissue types^7–9^.

Over the past few years, the Cancer Dependency Map (DepMap) database has gained significant popularity as a valuable resource for data mining in cancer research fields^10–13^. Hosted primarily by the BROAD Institute and collaboratively supported by the Sanger Institute, the DepMap database serves as a comprehensive repository of genome-wide high-throughput genetic knockout screening data. It offers systematic means to assess genetic dependencies for genes of interest.

The DepMap database incorporates diverse genomic data collections, including gene expression and mutation data. It houses genome-wide CRISPR screening data for approximately 1000 Cancer Cell Line Encyclopedia (CCLE) cell lines, enabling the derivation of CRISPR effect scores that measure the dependency levels on individual genes within each cell line. Behind the scenes, intricate data processing procedures have been developed and continually improved over time to process, normalize, and integrate data from multiple resources. These efforts ensure that the DepMap data is both comparable and meaningful, aiming to create a comprehensive dependency map for available cell lines^14,15^.

Our initial study of working with expression data and the earlier version of DepMap data^16^, has investigated the so-called “oncogene addiction” phenomenon that cancer cells are often addicted to (i.e., physiologically dependent on) the sustained activity of specific activated or overexpressed oncogenes for maintenance of their malignant phenotypes^17^. Along with findings from other researchers^4,18,19^, these studies have demonstrated the data mining potential of the DepMap database to explore and uncover biological themes underlying oncogene addiction and cancer dependency^16^.

Most studies evaluating the role of RAS genes in oncogenesis have focused on only a few specific RAS and RAS related genes or have been limited to the examination of a single tissue or tumor type^1–6^. Previous computational studies on oncogenic driver genes have predominantly relied on genomic mutation data^7–9^. The availability of datasets in DepMap database utilizing CRISPR effect scores to assess the cellular and growth impact of genetic disturbances on all individual genes across the genome provides a significant opportunity to obtain more systematic and direct evidence for the impact of RAS and RAS related genes in cell growth.

In contemporary cancer research, it has become increasingly common for researchers to present data mining outcomes derived from the DepMap database. This valuable resource contains CRISPR effect scores for genes of interest, which are often extracted for individual genes and then compared across tissue types or between mutant and wild-type (WT) cell lines. These scores are typically subjected to statistical analysis using native statistical methods such as t-tests.

The Limma method is a powerful and robust statistical approach that is specifically designed for high throughput data analysis^20^. Motivated by promising observations from comparisons of CRISPR effect scores for KRAS genes in RAS mutant and RAS WT lines, we utilized the Limma method to systematically analyze CRISPR effect score. To address the challenge of evaluating the prominent pervasive oncogenic role of RAS gene mutations across multiple tissue types within the context of genome-wide genes, we used Limma based methods on the DepMap dataset to enhance the statistical power.

In this report, we performed differential analysis of high-throughput CRISPR screening data by the limma method^20^. Our systematic analysis reveals essential genes for both RAS mutant and RAS WT lines across multiple tissue types from the DepMap database. We observed that RAS genes, specifically KRAS or NRAS, emerged as the most or nearly the most differential gene(s) between contrasts of RAS vs. WT lines in corresponding subsets of tissue types that we defined as KRAS- or NRAS-engaged tissue type also supported by other evidence from gene expression data and association analysis of gene mutation status and dependency data. Exhaustive computational screenings not only consolidated the observed results not likely occurring by random chances, but also provided additional insights for other oncogenic driver genes. To the best of our knowledge, this study represents the first instance of a data mining investigation utilizing high-throughput genome-wide dependency data, which provided substantiated evidence that mutated RAS genes serve as the prominent pervasive oncogenic drivers across a much broader spectrum of tumor types, extending beyond the traditionally recognized KRAS-associated tumors. Furthermore, our results shed light on the concept of tissue-specific permissiveness in the context of mutant K- or N-RAS oncogenesis.

## Methods

### Data preparation

Pathway map of RAS pathway annotated by RAS Initiative was downloadable and internally maintained from RAS central (https://www.cancer.gov/research/key-initiatives/ras/ras-central/blog/2015/ras-pathway-v2). The annotations of pathways and genesets were downloaded from MSigDB databases (https://www.gsea-msigdb.org/gsea/msigdb/). The Biocarta pathway signature used the Biocarta pathway gene lists from MSigDB from the Broad Institute (https://www.software.broadinstitute.org/gsea/msigdb/genesets.jsp?collection=CP:BIOCARTA) (note: the original Biocarta pathway collection database has been retired), which is licensed under CC BY 4.0 (https://creativecommons.org/licenses/by/4.0/) as described (https://www.gsea-msigdb.org/gsea/msigdb_license_terms.jsp). MSigDB gene sets derived from KEGG pathways are protected by copyright, (c) 1995–2017 Kanehisa Laboratories, all rights reserved. They are provided for use in the MSigDB collection under license to the Broad Institute, Inc., with qualified permission to include in this release as described (https://www.gsea-msigdb.org/gsea/msigdb_license_terms.jsp). Cell line meta data, CRISPR effect score data and genomic data of all available cell lines (RNAseq raw read count data from RSEM, mutation status data) for version 21Q1 (version 23Q2 data was also downloaded to validate the results of version 21Q1) all were downloaded from Cancer Dependency Portal( https://depmap.org/portal/) based on initial publications^10–12^. The proteome data was downloaded from pan-cancer proteomic map (ProCan-DepMapSanger) as a comprehensive resource available at https://cellmodelpassports.sanger.ac.uk. Three sets of driver gene data files are obtained directly from email communications with the authors^7,9^ or through downloading from controlled access (after application was approved) of International Cancer Genome Consortium (ICGC) Data Portal (https://dcc.icgc.org/) for The Cancer Genome Atlas (TCGA) part or for public ICGC part directly from URL designed for PCAWG: https://dcc.icgc.org/releases/PCAWG/driver_mutations referred by authors from previous study^8^.

### Data analysis

Data manipulation and analysis mostly was done using customized R scripts or existing R packages (www.r-project.org). CRISPR effect score data was assessed with utility functions of Bioconductor limma package for suitability of limma method^20^. Differential genes between RAS mutant vs. WT or KRAS mutant vs. WT cell lines were identified with topTable function after using lmFit function to set up the model with defined contrast matrix within the limma package^20^. Volcano plots and other assessment plots were made by either plot functions from limma package or by customized R codes. Differentially expressed genes from RNAseq data were identified using the Bioconductor packages DESeq2^21^ and edgeR^22^ to evaluate robustness and consistency of results. Built-in utility functions such as plotPCA or plotMDS were used to assess overall behaviors of the data. For DESeq2, except for that the read-count matrix was filtered using a customized minimal pre-filtering to keep only rows that have at least 10 reads total, follows all default settings and lfcShrink function was used for Log fold change shrinkage of the DEGs lists. For edgeR, the read-count matrix was filtered using the filterByExpr function with default parameters and samples were normalized using calcNormFactors(method = ‘TMM’) from edgeR. Differentially expressed genes (DEGs) were extracted using cutoffs at log2(fold change) > 0.58 and adjusted *P*-value < 0.05 (DESeq2) or FDR < 0.05 (edgeR). The corresponding DEG gene lists were collected and applied to customized R scripts that implemented the in-house developed pathway pattern analysis method called PPEP, which was previously described^23^. Briefly, gene lists were subjected to Fisher’s exact test-based pathway or gene set enrichment analysis using annotated databases including Gene Ontology (www.geneontology.org/), KEGG pathways (www.genome.jp/kegg/pathway.html) and MSigDB (http://software.broadinstitute.org/gsea/msigdb/index.jsp). The derived *p*-values were transformed by a formula of (-1)*log10(*p*-value) with all *p*-values less than 0.05 and *p*-values with the number of “hit” genes from the gene list for the corresponding pathway/gene set less than 2 were all converted to 0. The transformed *p*-value data matrix was used to derive pathway-level heatmaps using either customized R scripts that used pheatmap or gplots R packages, or the TM4 MeV tool from TIGR (mev.tm4.org/). In this study, we only focused on mutations of genes as the most common genomic changes for oncogenesis, and also have only focused on mutations that occurred at the coding regions of a gene (excluding silent mutation type) for the reasons discussed in the Discussion section.

To test if the behaviors of KRAS or NRAS gene in the differential analysis can be achieved by random chances, exhaustive computational screenings is performed (top panel of Supplementary Fig. 10). Briefly, for any of the qualified genes: i.e., gene X with mutation(s) in cell lines and with sufficient numbers of mutant and WT lines from each tumor type (number of samples in each group > = 3) (ranged from about 400 to 8000 mutated genes varied in different tissue types out of total ~ 17k genome-wide genes in CRISPR screening data), limma analysis was performed to derive differential genes between gene X mutant lines vs. gene X WT lines for CRISPR effect score data. Statistical metrics were collected from the procedure, from which corresponding *p*-values or probability statistics were derived.

To test if the behaviors of RAS (K-, N- and H-) gene in the differential analysis can be achieved by random chances, exhaustive computational screenings is performed. Briefly, computational screening of unique trials (*n* = 10000) for randomly selected 3 mutated genes vs. corresponding WT lines in each tumor type for differential genes of CRISPR effect scores were performed to derive differential genes between the mutant lines vs. corresponding WT lines for CRISPR effect score data. Statistical metrics were collected from the procedure, from which corresponding *p*-values or probability statistics were derived.

Within the DepMap database a CRIPSR effect score of 0 denotes a gene that is not essential whereas a score of -1 corresponds to the median of all common essential genes after normalizing and standardizing the data. Based on this fact, for each of the seventeen thousand genes in the CRIPSR effect score dataset, it was determined whether there exist significant association of the presence of mutations of this gene with the dependency on this gene denoted by its corresponding CRISPR effect scores. Briefly, enrichment level of each gene was assessed by Fisher’s exact test on a typical 2 × 2 contingency table created for all cell lines on mutation status of a gene versus the dependency status (whether the gene has mutation(s) or not versus whether it has a CRISPR effect scores < (-1.0) or not in each cell line). For some tissue types with limited samples (e.g., pancreas with limited sample size of RAS WT lines), Barnard test, which is a more powerful alternative of Fisher’s exact test, was used on the same 2 × 2 contingency table. Wilcoxon rank sum test and t-test was also performed for purpose of comparison. Multiple testing with Benjamini-Hochberg method^24^ was performed on the derived *p*-values from enrichment analysis, and those from Wilcoxon rank sum test and t-test and corresponding adjusted *p*-values were derived. Analysis was done globally across cell lines of all tissue types or done specifically in cell lines of each tissue type.

## Results

### Data mining of CRISPR effect scores in DepMap showed prevalent difference in RAS genes between mutant lines vs. WT lines but also revealed drawbacks of individual assessment of genes of interest

RAS mutations are commonly considered as oncogenic drivers in Lung (LUAD), Colon (COAD), and Pancreas (PAAD) tumors, we are fascinated by the idea that very likely they may be critical oncogenic drivers in additional tumor types. To evaluate the potential oncogenic role of mutated KRAS in a much broader spectrum of tissue types we evaluated CRISPR effect scores from the DepMap database. The CRISPR effect scores within the DepMap database’s CRISPR screening data offer insights into the dependency levels on corresponding genes within cell lines.

RAS genes rarely exhibit the most negative CRISPR effect scores (data not shown). This finding is consistent with the fact that the CRISPR effect score primarily reflects a gene’s ability to impact cell survival and proliferation, rather than its presumed oncogenic role. However, considering the context of oncogenesis, it is the genomic changes, particularly mutations, occurring in oncogenic genes, distinguishing them from other essential genes without such functions.

If RAS mutations confer a survival and growth advantage in cell lines, indicative of their oncogenic addiction nature, we hypothesized that RAS mutant cell lines would exhibit a greater dependency on these RAS genes (more negative CRIPSR effect scores) compared to RAS wild-type (WT) lines. Consequently, our primary focus was to assess whether the KRAS gene consistently displayed more negative CRISPR effect scores in KRAS mutant lines, as compared to WT lines, across various tissue types, rather than emphasizing the absolute negativity of the CRISPR effect scores for KRAS genes in these cell lines.

We initially focused on analyzing the CRISPR effect scores of the KRAS gene. Encouragingly, we observed a clear negative shift in the CRISPR effect score distribution of KRAS mutants compared to WT lines. Furthermore, we identified a significant difference in scores between KRAS mutant and WT lines across all tissue types that had sufficient sample sizes (Supplementary Fig. 1). According to the DepMap database, a more negative value of the CRISPR effect score for a gene indicates a higher dependency of the cell line on that specific gene^10–12^. Remarkably, our findings revealed that KRAS mutant lines displayed greater dependency on the KRAS gene than their wild-type counterparts across all tissue types. This dependency was evident not only in the tissue types conventionally associated with RAS genes, such as lung, colon, and pancreas, but also in numerous other tissue types examined.

We further extended our comparison to encompass all RAS mutant lines (including those with KRAS, NRAS, and HRAS mutations) versus WT lines, significantly broadening the tissue types included in the comparison. Interestingly, we consistently observed a negative shift in the distribution of KRAS CRISPR effect scores in RAS mutant lines compared to WT lines across many tissue types. However, we did observe instances in certain tissue types where the RAS mutant and WT lines exhibited little or no differences in the distribution of their KRAS scores (highlighted in red rectangles, Supplementary Fig. 2A). This observation was expected, particularly in tissue types like skin, where NRAS, not KRAS, is recognized as one of the main oncogenic players in skin cancer, such as melanoma, based on observed mutation patterns in cancer patients^1^.

Therefore, to better understand the role of RAS genes in these tissue types, we examined the CRISPR effect scores of the NRAS gene in comparison to RAS mutant versus WT lines within the same set of tissue types (Supplementary Fig. 2B). As anticipated, we observed a clear negative shift in the distribution of CRISPR effect scores of NRAS gene in RAS mutant lines compared to WT lines in tissue types that exhibited little or no differences in the KRAS scores (highlighted in red rectangles, Supplementary Fig. 2B). This finding indicated that in these tissue types, NRAS may compensate for the role of KRAS in oncogenesis, and NRAS, rather than KRAS, could play a major role in driving oncogenesis. These observations align with expectations based on the existing literature, which has reported the prevalence of RAS gene mutations in these tissue types^1,2,7–9^.

To evaluate statistical differences, we employed native t-tests, a commonly used method. For the KRAS gene, our analysis revealed that the majority of tissue types exhibiting dissimilar score distributions (Supplementary Fig. 2A) also demonstrated significant t-test *p*-values (red arrows, Supplementary Fig. 2A). Conversely, certain tumor types appeared visually distinct but did not yield significant t-test *p*-values (green arrows, Supplementary Fig. 2A). In contrast, when examining the NRAS gene (Supplementary Fig. 2B), we observed that numerous tumor types did not achieve significant t-test *p*-values (green arrows, Supplementary Fig. 2B), despite apparent trends.

These observations underscore an important consideration: the data analyzed in our study were generated using high-throughput technology. With the advancements in this field and the availability of specific statistical analysis methods tailored for high-throughput data, it becomes imperative to leverage the entire dataset rather than focusing solely on individual genes, as we did in this study. Employing a systematic approach that simultaneously analyzes all genes and data using statistically robust methods designed for high-throughput data is warranted. By adopting such an approach, we not only reduce the risk of overlooking relevant findings but also enhance the statistical power of our analysis. As described later in this manuscript, our efforts in this direction led to intriguing insights.

### Systematic analysis of high-throughput CRISPR effect data of DepMap revealed the prominent pervasive oncogenic role of RAS gene mutations and implication of tissue-specific permissiveness of mutant K- or N-RAS oncogenesis in a wide spectrum of tumor types

To perform a comprehensive analysis of the genome-wide (~ 17k genes) high-throughput CRISPR screening data from nearly 1000 cell lines, we opted to utilize the widely recognized linear model-based method called limma^20^. Limma, originally designed for high-throughput microarray data analysis, offers superior statistical power compared to the native t-test for high-throughput datasets by leveraging information from within-group replicates and borrowing information across genes^20^. This ensures that limma can effectively identify more significant findings from the CRISPR effect scores of CRISPR screening data obtained from the DepMap database.

Before proceeding with the limma analysis, we conducted an initial data assessment to ensure the suitability of applying the limma method to the genome-wide CRISPR gene effect score data from the DepMap database (Supplementary Fig. 3). Based on the robustness and flexibility of data distribution and types that limma method can be applicable to, the assessment confirmed that the CRISPR effect scores from DepMap are appropriate for analysis using the limma method.

Utilizing the limma method, we conducted an initial investigation to examine the differences in CRIPSR effect scores of genome-wide genes between KRAS mutants and WT lines. The results were presented visually using volcano plots, which effectively illustrate gene-level statistics (Supplementary Figs. 4 and 5).

As expected, KRAS emerged as the most significant gene, displaying a substantial difference at the adjusted *p*-value level, obtained through multiple testing (Supplementary Fig. 4). Additionally, KRAS exhibited the most pronounced negative logFC in comparisons between KRAS mutants and WT lines originating from lung and pancreas tissues (Supplementary Fig. 4). Consistent patterns were observed across other tumor types, with KRAS consistently identified as the most differential gene and the only gene exhibiting significant adjusted *p*-values through multiple testing. The only exception was noted in the Haemato_and_lymph (haematopoietic_and_lymphoid) category, where KRAS was identified as nearly the most differentially expressed gene (Supplementary Fig. 5). Overall, the limma analysis consistently ranked KRAS as one of the top one or two genes across almost all tumor types with sufficient samples (Supplementary Table 1).

To evaluate how other mutated genes behave in comparison to RAS genes, we did perform exhaustive computational screening for all qualified mutated genes, including commonly known oncogenic driver genes such as PIK3CA and BRAF as we did differential analysis for KRAS mutants vs. WT contrast, which confirmed that the results of KRAS mutant vs. WT contrasts were highly likely to be driven by the underlying RAS biology, not by random chance, and will be described in the next Results section.

Furthermore, we observed instances where native t-test *p*-values were not significant, but the adjusted *p*-values using the limma method yielded significance. This observation further highlights the advantages of employing high-throughput data analysis methods. Similar cases were also encountered during the comparison of RAS mutant and WT lines, as described in the subsequent analysis. This underscores the robustness and reliability of the limma method in detecting meaningful differences and potential insights that might be overlooked by traditional statistical approaches.

**Figure 1:**
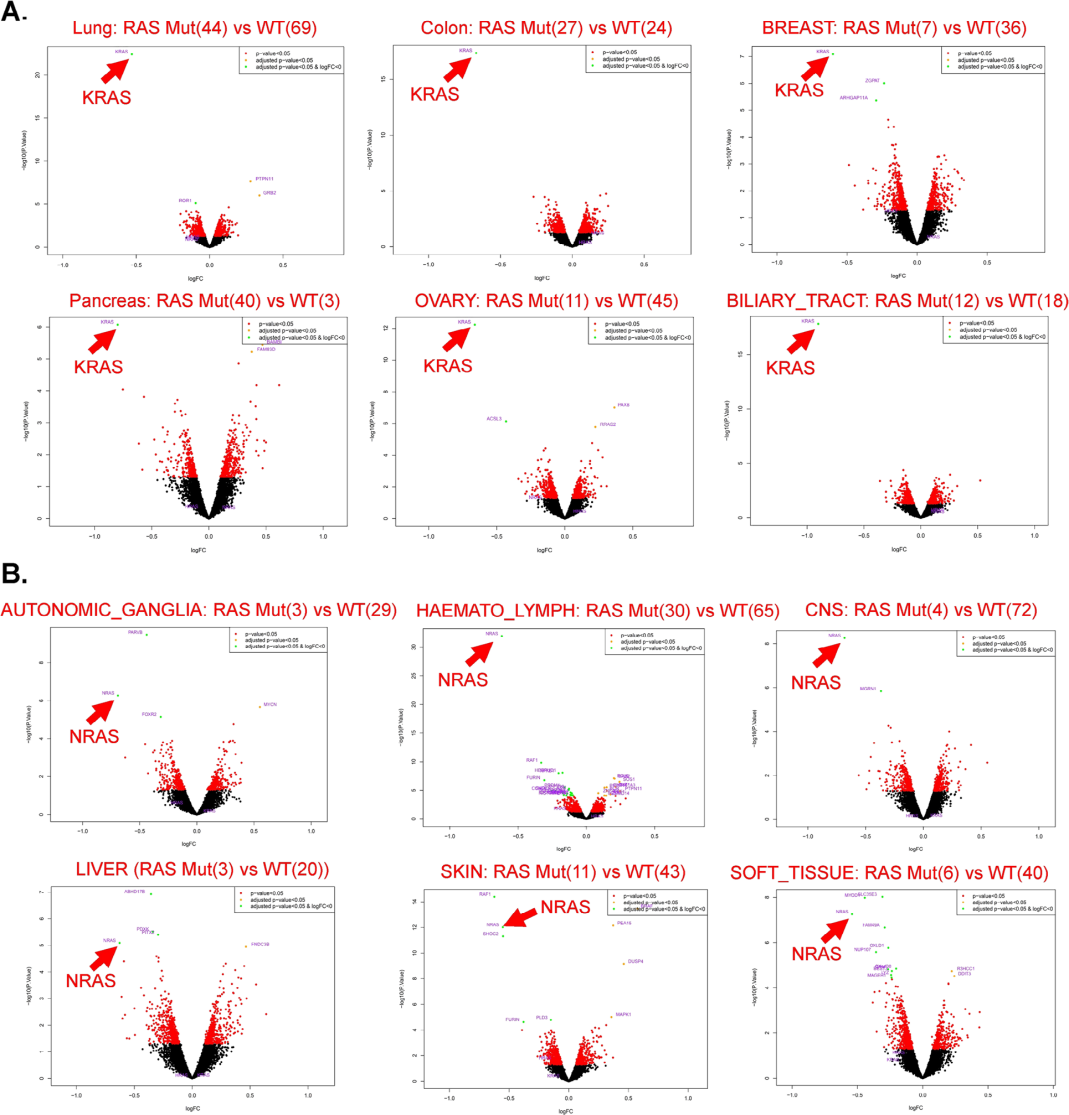
KRAS or NRAS was derived as the most or most nearly significantly differential gene for CRISPR gene effect at adjusted *p*-value < 0.05 with the most or nearly most negative dependency difference between RAS mutant vs. WT lines from corresponding subsets of tissue types. Volcano plots of all genes for CRISPR effect score data of DepMap showed KRAS as the most significantly or nearly the most significantly differential gene between RAS mutant vs. WT lines from a subset of tissue types including lung, colon, breast, pancreas, ovary, biliary tract (**A**) or NRAS as the most significantly or nearly the most significantly differential gene between RAS mutant vs. WT lines from another subset of tissue types including Autonomic Ganglia, Liver, CNS, Skin, Soft Tissue, and Haemato_Lymph (abbreviation for HAEMATOPOIETIC_AND_LYMPHOID_TISSUE) (**B**). Green data points: genes with significant adjusted *p*-value (< 0.05) for multiple testing and logFC < 0; orange data points: genes with significant adjusted *p*-value (< 0.05) for multiple testing and logFC > = 0; red data points: genes with significant raw *p*-value (< 0.05); black data points: genes without statistical significance. The parentheses after “Mut” or “WT” indicate number of mutant lines or number of WT lines, respectively. Note: limma model is set up on the whole dataset including all tumor types, and so all data is under the same roof of the limma model, by which power of the analysis was essentially increased as described earlier^20^. X-axis logFC: log2 fold change as for the actual difference of the CRISPR effect scores between RAS mutant vs. WT lines in volcano plots, since the values of CRISPR effect scores inherently in logarithm transformed scale were used directly in limma; y-axis –log10(p.Value): (-1)*log10 of raw *p*-value of limma analysis. Colon: LARGE_INTESTINE.

After obtaining highly significant results in the KRAS mutant versus WT contrast, we proceeded to investigate whether these observations would also hold for the RAS mutant versus WT contrast. RAS mutants encompassed all KRAS, NRAS, and HRAS mutant lines. Interestingly, in a similar analysis comparing RAS mutants to WT lines, the KRAS gene once again emerged as the most significantly differential gene in lung, colon, and pancreas (panel A of Fig. 1), which is consistent with the well-established role of KRAS as an oncogenic driver in these tissue types. With lung as an example (top left in panel A of Fig. 1), we observed that KRAS gene exhibited the most negative logFC and achieved the most significant adjusted *p*-values (< 0.05), suggesting that KRAS likely acts as an essential gene for RAS mutant lung cell lines (addicted to or dependent on as oncogenic addiction). Additionally, two other genes, PTPN11 and GRB2, were identified as significantly differential genes (adjusted *p*-values < 0.05) with positive logFC in the volcano plot, indicating their potential essentiality for RAS WT lung cell lines (top left in Panel A, Fig. 1). Notably, KRAS and these two genes are well-known critical components of the RAS pathway, as annotated by the RAS Initiative^25^ (Supplementary Fig. 6). These observations suggest that both RAS mutant and WT lines depend on RAS pathway genes as essential components.

However, what truly piqued our interest was the revelation that in numerous other tissue types, KRAS was also identified as the most or nearly the most significantly differential gene (adjusted *p*-values < 0.05) (Panel A, Fig. 1) beyond the main tissue types such as lung, colon and pancreas that RAS biology researchers commonly focus on. Intriguingly, for another distinct subset of tissue types, NRAS emerged as the most or nearly the most significantly differential gene (adjusted *p*-values < 0.05) (Panel B, Fig. 1).

Upon closer examination of the RAS mutation status in those tissues where NRAS was identified as the top differential gene, it became evident that most RAS mutant lines in these tissue types were, indeed, NRAS mutants. To corroborate these findings, we conducted differential gene analyses by directly comparing NRAS mutant versus wild-type (WT) lines across all examined tissue types, which yielded consistent results (see Supplementary Fig. 7). Taken together, these observations further support the prominent pervasive oncogenic role of RAS mutations.

Notably, in contrasts of NRAS vs. WT lines, lung tissue also featured NRAS as the most differential gene (Supplementary Fig. 7), despite the contrasts of KRAS mutant versus WT and RAS mutant versus WT lines consistently highlighting KRAS as the most differential gene in lung tissue (Supplementary Fig. 4 and Fig. 1). We also observed similar behavior from haematopoietic_and_lymphoid tissue type in that NRAS was derived as top differential gene in contrasts of NRAS versus WT (Supplementary Fig. 7) and RAS mutant versus WT lines (Fig. 1B), whereas KRAS as top differential gene in contrast of KRAS vs. WT lines (Supplementary Fig. 5). Given the fact that both lung and haematopoietic_and_lymphoid tissue types have sufficient numbers of mutated cell lines of either KRAS or NRAS, this suggested that there exists tissue-preference dependent mutation rate for KRAS or NRAS gene.

Inspired by the aforementioned differential gene analysis and the remarkable prominence of KRAS or NRAS as the top differential genes in subsets of tissue types, we classified tissue types into KRAS- or NRAS-engaged categories accordingly dependent upon whether KRAS or NRAS was derived as the top differential gene in corresponding tissue type. Subsequently, we conducted a direct assessment of the relationship between CRISPR effect scores for KRAS and NRAS within the context of KRAS- and NRAS-engaged tissue types and RAS mutation statuses. This analysis aimed to provide insights into the potential tissue-specific oncogenic capabilities of KRAS and NRAS mutations (Supplementary Fig. 8). A robust negative correlation was observed between the CRISPR effect scores of the KRAS gene and those of the NRAS gene, particularly under the context of RAS mutant cell lines either within KRAS and NRAS-engaged tissue types (top panel, Supplementary Fig. 8) or within all cell lines (data not shown; note: there are some tissue types in database that were not classified for their RAS engagement due to limited numbers of RAS mutant lines) when comparing to RAS wild-type (WT) cell lines. This behavior appeared to be attributed to the tissue type-specific deviation of CRISPR effect scores of mutated KRAS or NRAS genes from the WT lines in corresponding KRAS or NRAS-engaged tissue types (top panel, Supplementary Fig. 8A). Majority of NRAS and KRAS mutant lines formed two distinguished clusters separated from the main cluster of WT lines, which are consistent with their CRISPR effect scores of NRAS or KRAS gene tending to be more negative than those of WT lines (bottom panel, Supplementary Fig. 8A). This is consistent with the expectation that generally KRAS- or NRAS-engaged tissue types preferentially confer KRAS or NRAS-dependency of RAS mutants presumably through fostering their tissue-type specific mutation rates of KRAS or NRAS gene, respectively.

Notably, the “conversion” behaviors of four converted lines that shifted from the clusters of their original KRAS-engaged tissue types to those of NRAS-engaged tissue types (cell lines in blue triangles indicated by blue arrows at bottom panel, Supplementary Fig. 8A), were coincident with their acquired NRAS mutations that differ from the expected KRAS mutations that their original KRAS-engaged tissue types would favorably foster (Supplementary Fig. 8B). Similarly, the “conversion” behaviors of seven converted lines that shifted from the clusters of their original NRAS-engaged tissue types to those of KRAS-engaged tissue types (cell lines in red circles indicated by red arrows at bottom panel, Supplementary Fig. 8A), were coincident with their acquired KRAS mutations that differ from the expected NRAS mutations that their original NRAS-engaged tissue types would favorably foster (Supplementary Fig. 8C). In addition, the deviation of some HRAS mutant lines from the WT lines were also coincident with their HRAS mutation driven negative CRISPR effect scores of their HRAS gene, suggesting the likely role of HRAS like KRAS or NRAS once mutated despite of their very limited number of incidences in DepMap dataset (Supplementary Fig. 8D and 8E).

These compelling observations revealed that although endowed KRAS-engaged or NRAS-engaged tissue types would preferentially foster KRAS or NRAS mutations, respectively (Supplementary Fig. 8A), the bona fide acquired KRAS or NRAS mutations in those converted lines (Supplementary Fig. 8B and 8C) would override their original tissue type predisposition. Those findings not only supported the prominent pervasive oncogenic power of RAS gene mutations from another perspective, but also offered insights into the tissue-type specific permissiveness of mutant K- or N-RAS oncogenesis across a diverse spectrum of tumor types.

**Figure 2:**
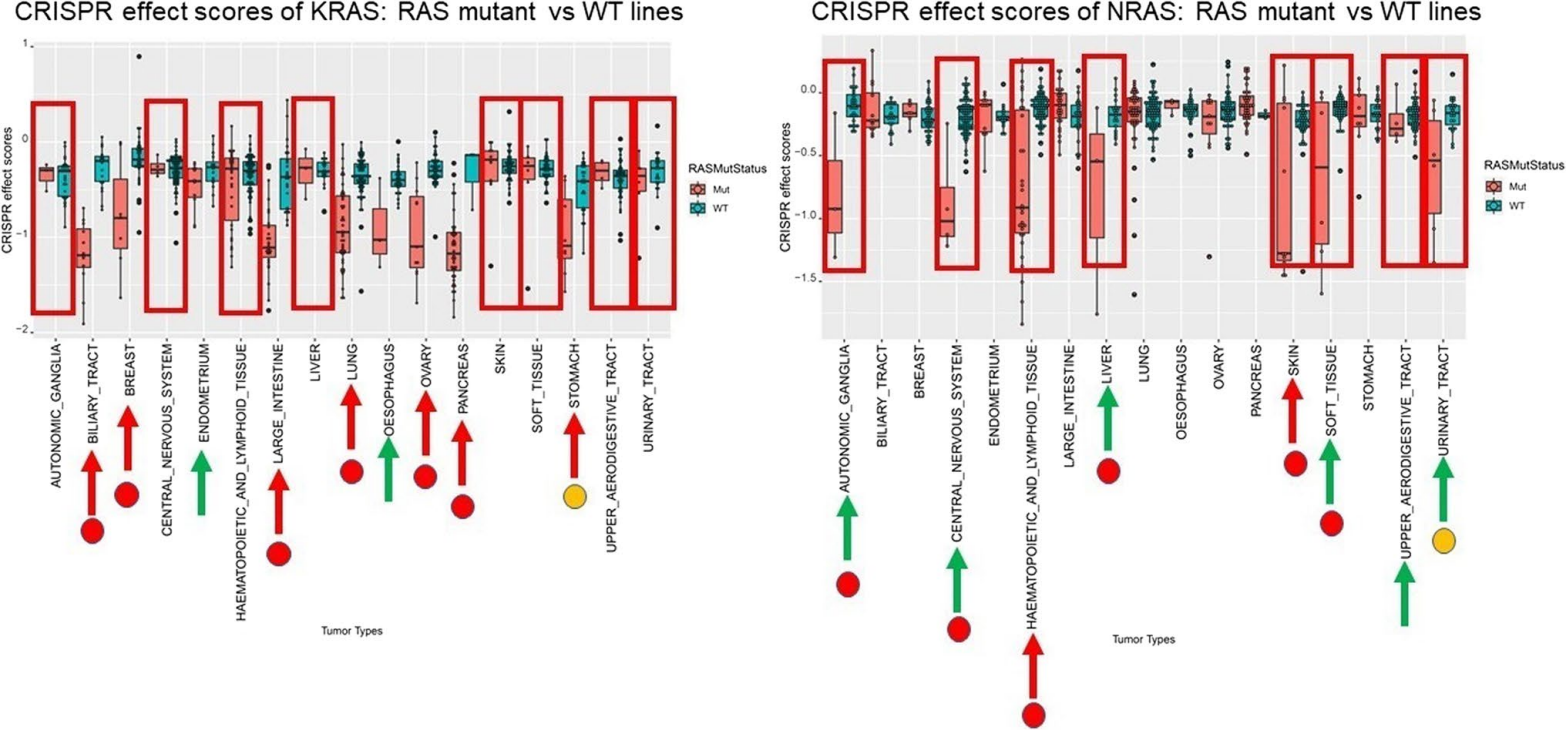
Largely improved statistical power and benefit using limma model comparing to commonly seen cherry-picking type of analysis of individual gene within the DepMap database. Comparison of CRIPSR effect scores in boxplots of KRAS (left) or NRAS (right) between RAS mutant vs. wild type (WT) lines from various tissue types in DepMap. Only tumor types from DepMap with at least 3 samples in both RAS (K-, N-, H-) mutant and WT lines would be used for comparison. This is similar to Supplementary Fig. 2 but here as highlighted by the additional red circles to show that the high throughput limma results of significant *p*-values with multiple testing essentially not only consolidate all native t-test results that are significant, but also make up many cases that were not significant by native t-test indicated by the green arrows. Also, in the cases of tissue types indicated by the yellow circles for stomach and urinary tract, where only limma raw *p*-value without multiple testing are significant. As shown in the volcano plots in Supplementary Fig. 9, although these two tumor types seem not calling RAS genes as significantly differential genes at level of adjusted *p*-value, however, as indicated by the red arrows in plots, KRAS or NRAS is still derived as the top differential gene at raw *p*-value level for stomach or for urinary tract respectively. Red arrow: native t-test *p*-value is significant; Green arrow: native t-test *p*-value is not significant. Note: CRISPR effect scores of each gene reflects the dependency level on the corresponding gene in the cell line, where the more negative the value of the CRISPR effect score is, the more likely this cell line is more dependent on this gene.

In summary, the limma analysis consistently revealed that across multiple tissue types with sufficient samples (Table 1), either KRAS or NRAS emerged frequently as the top and uniformly as one of the top 4 genes, reinforcing their significance in the context of RAS mutant versus WT lines. Even in tissue types where RAS genes were not ranked as the top differential genes, the top genes were still found to be RAS-related genes (Table 1). Furthermore, as highlighted in red in the summary table (Table 1), like the contrast of KRAS mutant lines versus WT lines described earlier, we observed more cases where the native t-test *p*-value was not significant, but the adjusted *p*-value using the limma method was significant for many tissue types. This finding suggests that the limma model can significantly improve statistical power and provide substantial benefits compared to commonly used cherry-picking analysis of individual genes within the DepMap database, underscoring the importance of systematically assessing high-throughput data. In fact, the limma results on high-throughput CRISPR screening data of DepMap not only consolidated all significant native t-test results but also redeemed many cases that were originally not deemed significant by native t-tests (Fig. 2).

**Table 1:** Summary of limma results including top differential genes and top genes’ statistics between RAS mutant vs. WT lines from various tissue types from DepMap database.

TumorTypes	Limma_TopGenes	Limma_Statistics	adjusted.*P*-Val	t-test_*P*-Values	Note
AUTONOMIC_GANGLIA	NRAS	Top 2 but most negative/adjp	0.005	0.176677483	
BILIARY_TRACT	KRAS	Top 1/adjP	2.61E-14	6.40E-07	only one gene with adj.*p* < 0.05
BREAST	KRAS	Top 1/adjP	0.001	0.038945017	
CENTRAL_NERVOUS_SYSTEM	NRAS	Top 1/adjP	9.73E-05	0.05326553	
ENDOMETRIUM	HCFC1R1(HPIP)	Top 1/adjP	0.025	0.004017588	Ras-related gene: Sci Rep.,2015,5:9429.
HAEMATOPOIETIC_AND_LYMPHOID	NRAS	Top 1/adjP	2.11E-28	2.85E-06	
LARGE_INTESTINE (Colon)	KRAS	Top 1/adjP	7.73E-14	1.68E-08	only one gene with adj.*p* < 0.05
LIVER	NRAS	Top 4 but most negative/adjP	0.036	0.331654472	
LUNG	KRAS	Top 1/adjP	6.68E-19	7.05E-11	
OESOPHAGUS	FANCI	Top 2/adjP	0.003	0.022969151	Ras-related gene: Onco Targets Ther. 2020, 13:451
OVARY	KRAS	Top 1/adjP	1.03E-08	0.00107161	
PANCREAS	KRAS	Top 1/adjP	0.015	0.041467891	
SKIN	NRAS	Top 4 with RAF1 and BRAF on top/adjP	4.17E-09	0.022067713	
SOFT_TISSUE	NRAS	Top 3/adjP	3.28E-04	0.115914845	
STOMACH	KRAS	Top 1/P.Value	0.077	0.01543102	Top1 DEGs at P-val = 5.24E-06 with nonsignificant adjusted.P-Val = 0.077
UPPER_AERODIGESTIVE_TRACT	RASSF8	Top 3/adjP	0.028	0.078226384	Ras-related gene: annotated in RAS pathway of RAS program
URINARY_TRACT	NRAS	Top 2/P.Value	0.144	0.085928442	Top2 DEGs at P-val = 1.63E-05 with nonsignificant adjusted.P-Val = 0.144; Top 1/adj.P.Val in NRAS mutant vs. WT

Interestingly, for the cases of stomach and urinary tract tissue types, although limma-derived raw *p*-values without multiple testing were significant, limma did not classify RAS genes as significantly differential genes at the level of adjusted *p*-values within these tumor types. However, both KRAS and NRAS were still identified as the top differential genes at the raw *p*-value level (top, Supplementary Fig. 9). Additionally, in the dataset of urinary tract, the contrast of NRAS mutant versus WT lines did yield NRAS the statistical significance of multiple tests with an adjusted *p*-value < 0.05 (bottom-right, Supplementary Fig. 9). Similar observation was made in dataset of stomach in contrast of KRA mutant versus WT lines ((bottom-left, Supplementary Fig. 9). Collectively, this indicates the putative prominent pervasive role of RAS mutations in the oncogenesis of a very wide spectrum of tumor types.

To gain a deeper understanding of the differential gene analysis results across all examined tissue types, we conducted a thorough examination of the significantly differential genes based on CRISPR effect scores between RAS mutant versus WT lines. We classified these differential genes as potentially essential genes for RAS mutant lines (2nd column of Supplementary Table 2) or as essential genes for WT lines (3rd column of Supplementary Table 2), depending on whether they exhibited more negative scores in RAS mutant lines or in WT lines, respectively. As anticipated, many of these genes are well-known oncogenic genes from the RAS pathway, as annotated by the RAS Initiative (Supplementary Tables 2 and Supplementary Fig. 6). In addition to KRAS and NRAS, several other genes were identified as essential for RAS mutant lines in multiple tissue types, such as RAF1 and SHOC2, particularly in lymph/blood and skin tissues. Conversely, other genes like BRAF, SOS1, MAPK1, and GRB2 were essential for WT lines, with PTPN11 (i.e., SHP2) previously described as essential for WT lines in both lung and lymph/blood tissues (Supplementary Tables 2 and Supplementary Fig. 6).

### Exhaustive computational screening demonstrated the observation of KRAS, NRAS, or RAS gene as the top gene in differential analysis not possibly occurring by random chances

Our differential gene analysis of CRISPR effect data in contrasts of KRAS vs. WT lines consistently identified the KRAS gene as the top differential gene across numerous tissue types, extending beyond the three main tissue types (i.e., lung, colon, pancreas) traditionally associated with KRAS-driven oncogenesis. To assess the possibility of these occurrences being random chances for any qualified mutated genes with sufficient cell lines harboring mutations of the corresponding gene in each tissue type that we analyzed KRAS gene in differential analysis, we conducted exhaustive computational screening of all qualified mutated genes, following the procedure described in the methods section and outlined (Supplementary Fig. 10A). The statistical results of our screening are summarized (Supplementary Fig. 10). The main theme of the findings indicated that the observed significance of KRAS cannot be attributed to random chances for any other existing mutated genes in the tissue types that we analyzed KRAS in differential analysis (Supplementary Fig. 10B). This strongly suggests that the observed results are most likely driven by the underlying RAS biology.

Importantly, through these exhaustive screenings, we identified several interesting, mutated genes (column Top2Genes in the table of Supplementary Fig. 10B) that exhibited similar behavior to KRAS. When comparing KRAS mutant lines with WT lines for these genes, the limma method used in the computational screening also ranked these corresponding genes as the top differential genes. Interestingly, many of these genes are already well-known in the field, such as BRAF, PIK3CA, TP53, and CTNNB1, which are established oncogenic driver genes or tumor suppressor genes, as identified by recent computational studies on oncogenic driver genes^7–9^. Some of these genes, such as BRAF, PIK3CA, and CTNNB1, appeared across multiple tissue types (column Top2Genes in the table of Supplementary Fig. 10B). These findings suggest that amongst the top differential genes identified in these exhaustive screenings, even those that may not be familiar to us, but exhibit similar behaviors to KRAS and other well-known oncogenes, are likely to be novel oncogenic driver genes with biological relevance that was previously unknown. However, it is important to emphasize that in terms of their prevalence across tissue types, these mutated genes do not appear to be nearly as prominent and pervasive as the KRAS gene, which is consistently ranked as the top differential gene in not just one or two, but nearly all tissue types. This distinction sets the KRAS gene apart from the other top genes identified in the exhaustive computational screening.

For similar purpose, to test if the behaviors of NRAS gene as the top gene in the differential analysis can be achieved by random chances, similar exhaustive computational screenings were performed to assess the possibility of these occurrences being random chances for any qualified mutated genes in each tissue type that we analyzed NRAS gene in differential analysis. The statistical results of our screening (Supplementary Fig. 10C) are indicative of a similar theme of the findings that the observed significance of NRAS cannot be attributed to random chances for any other existing mutated genes in each tissue type that we analyzed NRAS gene in differential analysis, which again suggests our observed results are most likely driven by the underlying RAS biology.

Like the results obtained from the KRAS or NRAS mutant vs. WT contrast, the findings from the RAS mutant vs. WT contrast revealed even more pronounced observations, with RAS genes (i.e., KRAS or NRAS) consistently emerging as the top differential genes across a broader range of tissue types. To evaluate the possibility of achieving these results by random chances for any combination of three qualified genes with sufficient numbers of mutant cell lines, we conducted a similar computational screening procedure. In this procedure, we performed 10,000 trials of randomly selected combinations of three qualified mutated genes (ensuring sufficient sample sizes in each group of contrast) for each tissue type, resembling the three mutated RAS genes (K-, N-, H-) used in the RAS mutant vs. WT contrasts. The obtained statistics, summarized in Supplementary Tables 3 and 4 for two independent sets of unique trials for each tissue type, consistently indicated that the observed results for RAS mutants could not have occurred by random chances for any combination of three mutated genes within the DepMap database. This further supports the notion that the findings obtained from RAS mutant vs. WT contrasts are highly likely to be driven by the underlying RAS biology.

Furthermore, we also identified several mutated genes (column Top4Genes in Supplementary Tables 3 and 4) that exhibited behavior like RAS genes in one or up to three tissue types. Among the identified genes, several well-known oncogenic driver genes such as ALK, BRAF, PIK3CA, PIK3R1 (components of PI3 kinase), and CTNNB1 were recognized, aligning with recent computational studies on oncogenic driver genes^7–9^. Additionally, certain genes, including BRAF and PIK3CA, emerged in both sets of unique trials, not only in a single tissue type but across multiple tissue types, providing further support for their potential oncogenic roles. Similarly, less commonly known genes like WRN were also identified in both independent sets of unique trials (Supplementary Tables 3 and 4), consistently appearing in the same tissue types (i.e., colon, ovary, and stomach), with multiple occurrences in most cases, except for the first set of unique trials for the stomach (Supplementary Table 3). These findings collectively underscore the potential significance of these identified genes as novel oncogenic drivers, deserving further investigation and exploration.

To evaluate the biological significance of the differential genes obtained from the unique trials of the computational screenings, we assessed the enrichment levels of oncogenic driver genes within these genes. Specifically, we examined the enrichment of well-annotated driver genes, either defined by tumor type (green rows in Supplementary Table 5) or not defined by tumor types (green rows in Supplementary Table 6), in the differential gene lists derived from the unique trials (*n* = 10,000) of the computational screenings.

Remarkably, we observed significant enrichment of these well-annotated driver genes in most tissue types that had at least two differential genes identified from the unique trials. This finding is particularly encouraging, considering that the annotated driver genes were primarily derived from pioneering computational studies in the field^7–9^. Furthermore, we noted the presence of noticeable driver genes even in tissue types with only two differential genes (Supplementary Tables 5 and 6), further emphasizing the biological relevance and potential significance of these genes. Overall, the enrichment analysis provides compelling evidence supporting the notion that the identified differential genes from the unique trials of the computational screenings are biologically meaningful and potentially represent novel oncogenic drivers.

However, as described above, although many other oncogenic driver genes were revealed from these computational screening analyses, there is no single mutated gene that demonstrated the strength and breadth impact across multiple tissues as KRAS and NRAS. Once mutated, they behaved not nearly close to what mutant KRAS or NRAS behaved in each tissue type and consistently across tissue types. Only KRAS or NRAS was revealed as top differential genes consistently across multiple feasible tissue types with sufficient numbers of samples, whereas any other mutated genes only can do the same in one or two tissue types at the best they can do, as evident from the exhaustive computational screening results. These observations provide substantial support from another perspective for the prominent pervasive oncogenic role of RAS gene mutations.

### Enrichment and association analysis demonstrated oncogenic driver genes including RAS genes as the top genes out of genome-wide genes with the most significant association between the presence of mutations in a gene and dependency on this corresponding gene

According to the DepMap database portal (https://depmap.org/portal/), a CRISPR effect score of 0 indicates a non-essential gene, while a score of -1 corresponds to the median of all common essential genes, after data normalization and standardization. We conducted a comprehensive evaluation of over 17 thousand genes in the effect score dataset, assessing the significant association between the presence of mutations in each mutated gene and its corresponding dependency, denoted by CRISPR effect scores, within nearly 1000 cell lines from the DepMap database. The enrichment level of each gene was assessed using Fisher’s exact test on a typical 2 × 2 contingency table, as described in the methods section in more detail. Notably, KRAS and NRAS emerged as the top 1 and 2 gene respectively, along with other well-known oncogenic driver genes, within the list of genes exhibiting the most significant association. These findings were corroborated by the results of Wilcoxon rank sum test and t-test (Supplementary Table 7).

Remarkably, many of the top genes with significant Enrichment.Adjusted.p.Val were annotated as oncogenic driver genes in the literature (Supplementary Table 7), although many oncogenic driver genes in the top list did display significant enrichment levels when assessing individual genes and strikingly only a handful of oncogenic driver genes had the significant enrichment after multiple testing-correction. The significance levels of Enrichment.Adjusted.p.Val for KRAS and NRAS also displayed quite a dominance over any other oncogenic driver genes including BRAF and PIK3CA in the top list. Interestingly, the vast majority of significant oncogenic driver genes on the top list are exclusively from the RAS signaling pathway (except for CTNNB1). This suggests that RAS signaling may be a special case with RAS genes as the top genes having the prominent and pervasive effect on reprogramming cells to become dependent on the oncogenic changes. On the other hand, these observations are consistent very well with, if do not directly support, the unique and exceptional behaviors of RAS (K- or N-RAS) genes over any other oncogenic driver genes in the findings that RAS genes were revealed as the only genes with prominent and pervasive oncogenic roles from the exhaustive computational screening analysis results (Supplementary Fig. 10, Supplementary Tables 3 and 4).

These results suggest that KRAS exhibits the most significant association between the presence of its mutations and its dependency across all tissue types. Moreover, we sought to verify if this observation holds true for each specific tissue type. As expected, the results for colon and lung consistently identified KRAS as the top gene with the most significant enrichment and association (top and middle tables in Supplementary Fig. 11). Although pancreas did not yield a significant enrichment result, likely due to the limited sample size of RAS WT lines, both Wilcoxon and t-test revealed significant association with KRAS (circled in red, bottom table in Supplementary Fig. 11). Additionally, the more powerful Barnard test, an alternative to Fisher’s exact test, indicated a significant *p*-value of 0.03 for KRAS in pancreas (data not shown).

Interestingly, we also observed significant enrichment and associations for NRAS or KRAS within tumor types such as blood, lymphoid, and ovary (Supplementary Fig. 12). In other tissue types, while not significant at multiple test levels, KRAS and PIK3CA consistently appeared as the top genes with the most significant enrichment and association at the raw *p*-value level (Supplementary Fig. 12). Similarly, in several other tissue types, NRAS consistently emerged as the top gene with the most significant association, either at the multiple test level in green rows or at the raw *p*-value level (Supplementary Fig. 13). These findings not only support the prominent pervasive oncogenic role of RAS mutations, but also are in line with the concept of KRAS or NRAS engaged tissue types derived from differential gene analysis of CRISPR data described earlier.

### Other genomic data supports the findings from differential gene analysis of CRISPR screening data

We identified numerous oncogenic driver genes from the analysis of CRISPR screening data of DepMap. To reinforce these results, we extensively searched for supporting evidence from other genomic data of DepMap, including the mutation status of the cell lines. Among the identified essential genes, we focused on BRAF as a proof of concept. BRAF was identified as the top differential gene for CRISPR effect scores between RAS mutant and WT lines, and as an essential gene for WT lines from skin origin (Supplementary Table 2).

**Figure 3:**
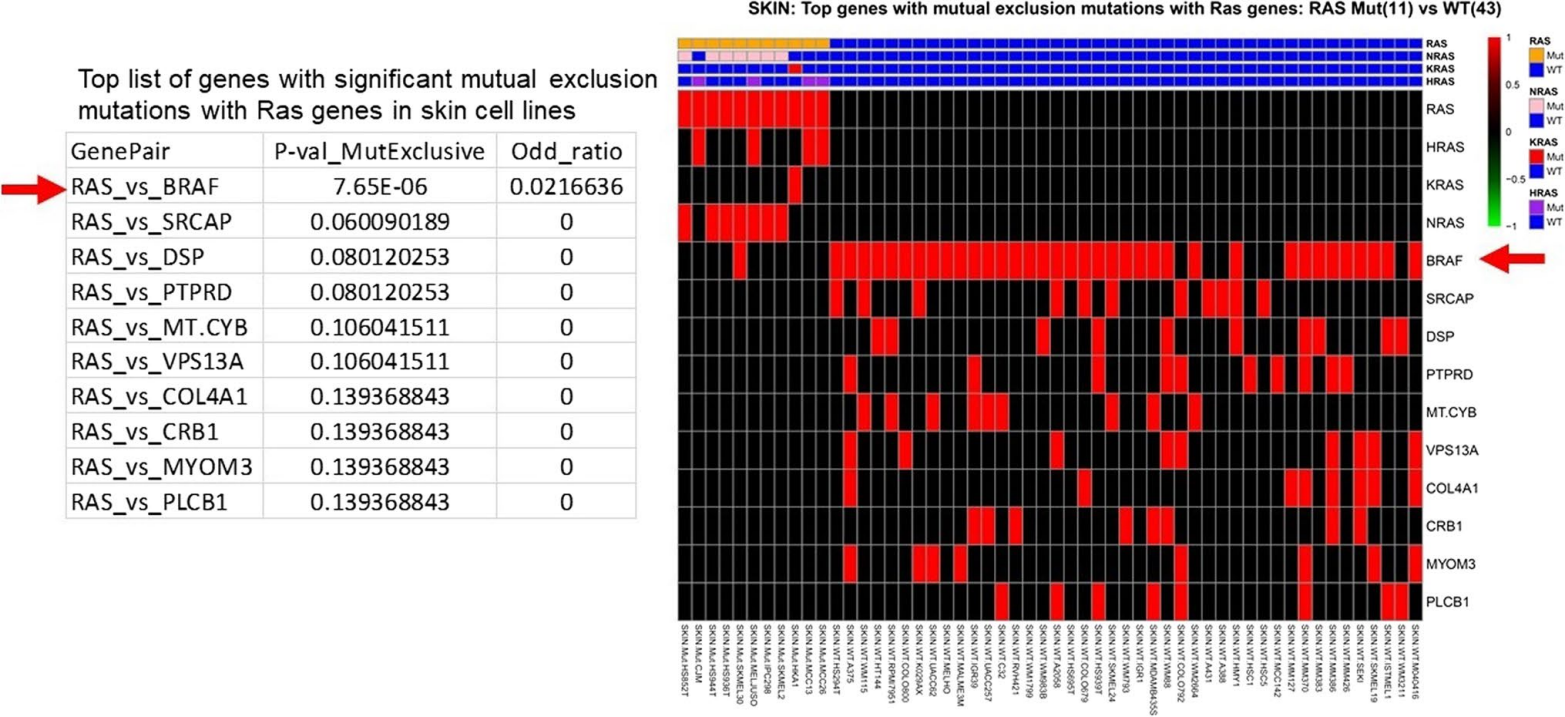
BRAF was revealed as an essential gene for RAS WT lines with the most significant mutual exclusive mutations between RAS genes in skin cell lines. Left panel: Top list of genes with mutual exclusion mutations with RAS genes in skin cell lines. Right panel: heatmap of mutation status from Top list of genes with mutual exclusion mutations with RAS genes in skin cell lines. Mutual exclusion mutation assessment was done by Fisher’s exact test with 2 × 2 contingency table created for all cell lines (whether a cell line has a mutation of this corresponding gene or not versus whether it has RAS mutation(s) or not), which was used to assess the significance of mutual exclusion.

Further assessment and exploration of mutually exclusive mutated genes with RAS genes in skin cell lines revealed that BRAF was the top gene exhibiting a mutual exclusive mutated pattern with RAS genes (Fig. 3). As BRAF is a well-known oncogenic driver gene for skin cancer (e.g., SKCM) like RAS genes, these findings from the mutation data strongly supported our observation that BRAF is one of the essential genes in RAS WT skin cell lines, as identified through differential gene analysis from CRISPR effect scores. Similarly, in colon cell lines, the assessment and search for mutually exclusive mutated genes with RAS genes also highlighted BRAF as the top gene exhibiting a mutual exclusive mutated pattern with RAS genes (Supplementary Fig. 14), consistent with earlier reports^26,27^. While the differential gene analysis of CRISPR effect score data did not detect BRAF as a significant gene at the level of adjusted *p*-value in colon, it was detected at the level of raw *p*-value as 0.0047 through limma analysis in colon (Supplementary Fig. 14, Supplementary Table 2, detailed result for BRAF in colon not shown). Together, these lines of evidence reinforce the notion that RAS WT lines also rely on the RAS pathway or RAS-related oncogenic genes such as BRAF.

In addition to CRISPR screening data, DepMap provides gene expression data or RNAseq data for the cell lines and we did obtain proteome data outside DepMap database for those CCLE cell lines. We investigated both RNAseq and proteome data at high-level using technique of dimensional reduction including MDS and PCA that would represent the main trend of expression data (Supplementary Fig. 15). Both RNAseq and proteome data revealed overall difference in transcriptional profiles between cell lines from KRAS-engaged tissue types highlighted in a large red circle versus NRAS-engaged tissue types highlighted in a large blue circle (left panels of A and B, Supplementary Fig. 15), which supported the idea of KRAS- or NRAS-engaged tissue types that inferred from the differential analysis of DepMap CRISPR effect data.

However, both RNAseq and proteome data suggest that the impact of RAS mutations on the difference between (K- or N-) RAS mutant vs. WT lines could be weaker comparing to the tissue-specific expression profiles, or such impact would confer through other mechanisms such as signaling or post-transcriptional modifications that may be explored using other omics data. (Supplementary Fig. 15). In addition, even for those from the same tissue origins, there are large variations amongst the transcriptional profiles of those converted lines (with labels in plots, described in Supplementary Fig. 8B, 8 C) with acquired KRAS or NRAS mutations that are different from what their original RAS engaged tissue types would foster. These observations suggested that there is also a possibility that other mechanisms such as signaling at protein levels rather than transcriptional changes would also likely be involved. Unfortunately, we do not have post-transcriptional modifications data for these CCLE cell lines available from DepMap database to address this possibility.

**Figure 4:**
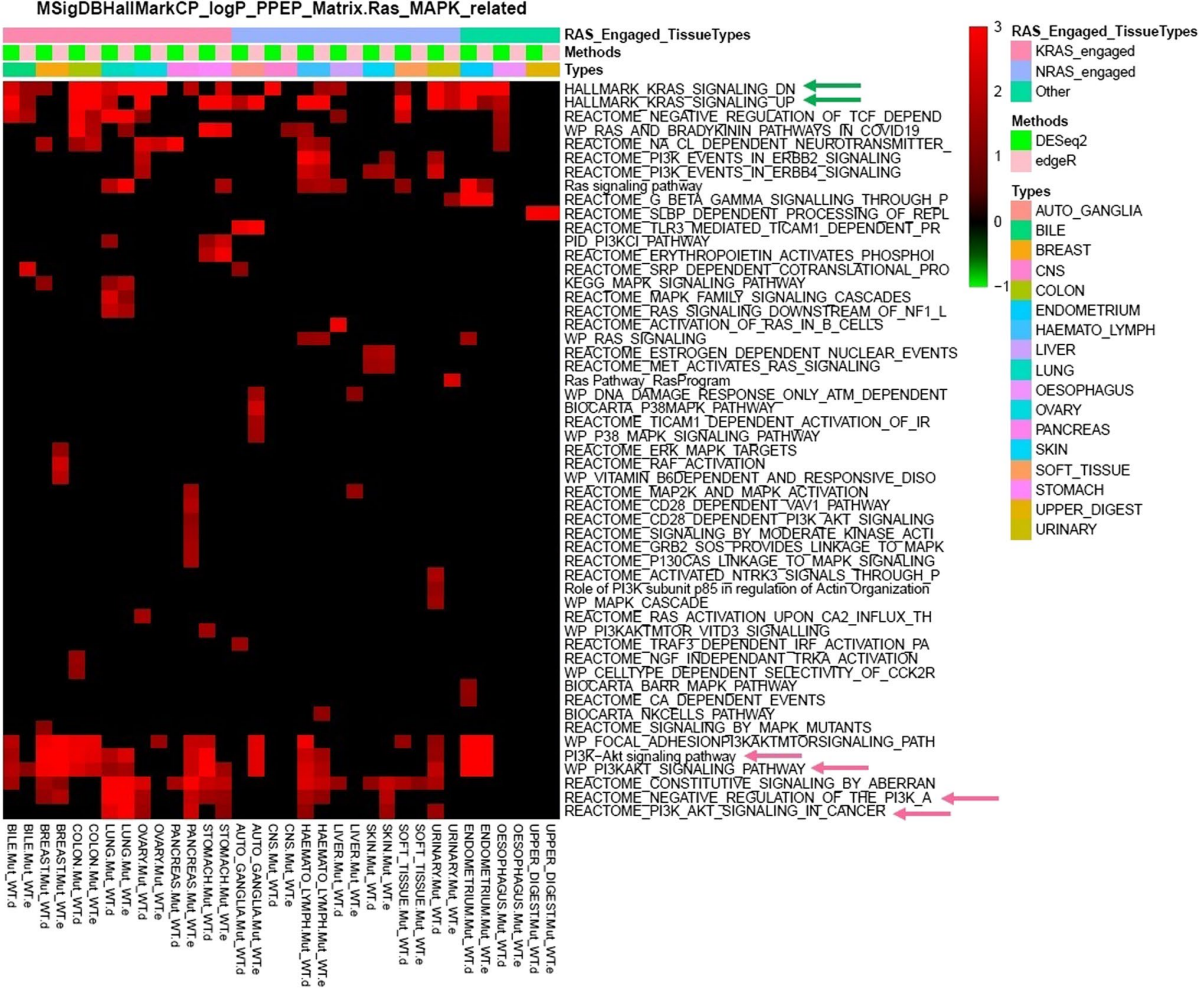
Hallmark KRAS signaling and PI3K signaling pathways were preferentially enriched in differentially expressed genes between RAS mutant vs. WT lines across multiple tissue types derived from RNAseq data of DepMap database. After derived the differentially expressed genes between RAS mutant lines vs. WT lines in various tumor types that have matched samples with CRISPR screening data using two RNAseq analysis methods (edgeR and DESeq2), the in-house PPEP^23^ analysis was performed to assess how pathways are enriched in these differential gene lists across multiple tissue types. KRAS signaling and PI3K signaling pathways from various sources of pathway/geneset annotations from MSigDB database enriched widely across differential gene lists were indicated by green and pink arrows, respectively. Types: tissue types; Two methods used for deriving DEGs: e: edgeR; d: DESeq2; Annotations of RAS_Engaged_TissueTypes are based on the top differential gene as KRAS, NRAS, or genes of RAS pathway between the contrasts of KRAS, NRAS and RAS mutant vs. WT lines in each respective tissue type for differential analysis of CRISPR effect score data.

Observing a large difference in transcriptional profiles between tissue types, we extracted the differentially expressed genes between RAS mutant lines and WT lines in each individual tissue type that had matched samples with CRISPR screening data. Subsequently, we utilized an in-house pathway pattern extraction pipeline (PPEP) to assess pathway enrichment in these differential gene lists across multiple tissue types (Fig. 4). As expected, our analysis revealed widespread enrichment of the KRAS signaling and PI3K signaling pathways across many tissue types (Fig. 4). This strongly suggests that the expression profiles of these RAS mutant cell lines may have undergone rewiring, likely triggered by the RAS mutations as corresponding genetic alterations, and adapted to promote the fitness of RAS mutants, thereby potentially conferring the oncogenic benefits associated with RAS gene mutations for oncogenesis.

### Robustness of the findings was supported by a more recent DepMap dataset and data mining of computational studies on cancer driver genes

An important factor to consider is the periodic updates made to the DepMap database, which occur on a quarterly basis. Given the significant time interval between our initial analysis and the submission of this manuscript, we made the deliberate choice to utilize the most current version available at that time, specifically version 23Q2, to consolidate our results derived from the older version 21Q1 of the DepMap data. Employing this updated dataset, we conducted a comprehensive limma analysis aimed at identifying differential genes for CRISPR effect score data between RAS mutant and WT lines (as illustrated in Supplementary Figs. 16 and 17). Notably, our findings remained remarkably consistent with our earlier results, providing further validation of the robustness and confidence in our observations.

Numerous studies have explored cancer driver genes using mutation data from diverse tumor types^7–9^. Although these studies do not specifically focus on RAS genes but generally on any generic oncogenic driver genes, the detailed data mining of the resulting oncogenic driver genes from these studies revealed that RAS genes were computationally predicted to be oncogenic driver genes across many tumor types (Supplementary Fig. 18). However, their findings neither have revealed RAS genes as the prominent or pervasive oncogenic driver gene distinct from other oncogenic genes (data not shown) due to the nature of their study and data sources and differences in methodology, whereas our study consistently showed in multiple threads of evidence for the prominent pervasive behavior of RAS genes in a diverse range of tissue types. In addition, our study extends beyond this by providing more precise and delineated insights into the oncogenic roles of RAS genes, specifically KRAS or NRAS, as the prominent oncogenic drivers within specific subsets of tissue types. This highlights the tissue-specific permissiveness and preference of mutant K- or N-RAS oncogenesis. Likely, these studies^7–9^ were limited by using only mutation data and protein structure information to make inferences. In contrast, our study distinguishes itself as the first to unveil insights derived from the analysis of genomic data and high-throughput gene dependency data of DepMap, which is more relevant in the context of this study. This distinction adds substantial confidence in terms of the data types and resources used, differentiating it from these primarily mutation data-based computational studies^7–9^.

## Discussion

Despite the high frequency of observed KRAS gene mutations, primarily in pancreas, lung, and colon cancer, RAS (K-, N-, H-) gene mutations are also widely prevalent in many other cancer types, which led to the notion that they contribute to oncogenesis in approximately 20% of human cancers. While the NCI RAS Initiative has made significant strides as a community-supported national effort, there remains a critical need to gather compelling evidence to substantiate the long-held notion that RAS gene mutations play a critical role as oncogenic drivers, influencing a much wider spectrum of cancer types beyond the main ones currently under the focus of RAS researchers.

In this report, we conducted a comprehensive and systematic differential analysis of high-throughput CRISPR screening data using the limma method. This rigorous approach led us to reveal RAS genes (KRAS and NRAS) as the most significantly differential genes between the (K-, N-, combined) RAS mutant versus WT lines across a wide spectrum of tissue types within the entire genome-wide dataset. Notably, our discoveries extended beyond the traditionally studied tissue types associated with RAS genes in oncogenesis, encompassing lung, colon, pancreas, and skin. Such behaviors of RAS genes are exactly the nature of oncogenic driver genes: once mutated, the cells become more dependent or more addicted to the mutated gene reflected by their CRISPR effect scores, which is in line with the proposed notion of oncogene addiction^17^. More importantly, our exhaustive computational analysis of mutated genes pointed out the prominent perversive oncogenic role of RAS gene mutations to a level that other oncogenic driver genes do not achieve. Our observations from differential gene analysis supports the prominent and pervasive oncogenic role for RAS gene: prominent in a way that RAS gene is the top gene in individual tissue type, and pervasive in a way that RAS gene is the only top gene common across multiple tissue types.

Moreover, our study provided intriguing insights into the potential distinct oncogenic roles of KRAS and NRAS in specific, corresponding subsets of tissue types. These observations suggest that either mutant KRAS or mutant NRAS could act as the primary oncogenic driver genes in distinct subsets of tissue types, which we denoted as KRAS-engaged or NRAS-engaged tissue types, respectively. Interestingly, mutant KRAS or mutant NRAS may also exert oncogenic influence within the same tissue types, such as lung. These findings offer compelling evidence pointing towards the potential tissue-specific preference and permissiveness of mutant K- or N-RAS oncogenesis and suggested that the (K- or N-) RAS gene mutations that may determine the tissue type-specific oncogenic capacity although preferentially cooperating with the revealed KRAS- or NRAS-engaged tissue types (Supplementary Fig. 8). However, it is crucial to acknowledge that our analysis was not able to fully assess the oncogenic role of HRAS as we did for KRAS or NRAS, mainly attributable to the constraints imposed by the limited availability of HRAS mutant cell lines representing specific tissue types within the DepMap datasets. Nevertheless, we think it is still reasonable to anticipate a similar behavior of HRAS as K- or N-RAS, given the observed similar trend for HRAS in Supplementary Fig. 8D and the common notion on how close HRAS is related to KRAS and NRAS as one of RAS gene isoforms.

Remarkably, our analysis consistently identified RAS genes as the top differential genes in CRISPR effect scores when comparing RAS mutant vs. WT lines across the majority, if not all, tissue types. In contrast, other oncogenic driver genes only appeared as top differential genes in one or a few tissue types when comparing their mutations to WT lines. The exhaustive computational screening and validation tests revealed that many of the oncogenic driver genes we identified were limited to one or two tissue types, in stark contrast to the pervasive behavior of RAS genes.

Furthermore, despite the potential differences in genetic background, metabolism, and epigenetic regulation between RAS mutant and WT cell lines, we consistently observed RAS genes occupying the top positions among the differential genes across various tissue types. The use of the limma method, specifically designed for high-throughput data, and the comprehensive analysis of the vast DepMap dataset, consisting of 17 thousand genes and nearly 1000 cell lines, made our findings highly unlikely to occur by chance. These consistent observations suggest that RAS gene mutations play a prominent pervasive oncogenic role, regardless of the tissue type.

It is worth noting that while RAS gene mutations may be prevalent in a wide variety of tissue types, there are other commonly mutated genes, such as TTN, with much higher mutation frequencies than RAS genes in many tissues (data not shown). However, these genes did not exhibit the same consistent and widespread oncogenic behavior as RAS genes in our study; instead, they appeared to function more as potential biomarkers for tumor mutation burden^28^. It is also worth noting that it is unknown whether the mutations present in the mutant cell lines that were included in this study have a biological effect on given genes carrying these mutations, although we did not use any tools to evaluate the biological impacts of the mutations as well as whether the mutation frequencies would be significantly above background. One reason is that current mutation impact assessment tools are still not at optimum and the tools assessing whether the mutation frequencies were significantly above background for a given gene would still have debatable uncertainty of association with oncogenesis. On the other hand, these tools may reduce the number of tissue types with feasible mutated genes that were needed to run much global analysis with more tissue types and more mutated genes as we wish for the exhaustive computational screenings. Consequently, we had chosen a more liberal route to account for all potential mutations at gene coding regions (excluding silent mutation type) to perform the exhaustive computational screenings. Such a choice was intended to be beneficial for computational screenings as well as the needed consistency with other analyses performed in this study.

Survey studies on RAS mutations have also emphasized the remarkable potential of RAS genes as oncogenic drivers in various tumor types^1,2,4^. However, our study provides robust evidence derived from experimentally measured genetic dependency data, directly capturing the cellular response and consequences of treated cells. Such data carry greater biological relevance than static mutation data alone^7–9^. While considerable efforts have focused on exploring the phenomenon of “oncogene addiction”, wherein tumors heavily rely on the sustained expression and activity of a single aberrantly activated oncogene, and appear to depend on epithelial differentiation status^16,29^, our current report elevates this notion to the next level. We propose that RAS genes play a prominent pervasive role in oncogenesis beyond merely being associated with “oncogene addiction”. While numerous oncogenes could participate in so-called “oncogene addiction”, in one or two tissue types or limited cellular contexts, our findings strongly suggest that only RAS genes hold this prominent and universal position in a wide range of tumor types examined.

The study mainly focused on gene mutations for their impacts on the dependency of RAS genes as well as other genes; However, it was known that gene mutation is not the only genomic change that would lead to oncogene addiction. Other genomic changes such as copy number alterations (CNAs) although much rare in general compared to gene mutations, would have similar impacts as mutations. However, due to limited cell lines and much lower occurrence of other genomic changes in the DepMap datasets as well as the potential to make the data interpretation more complicated, we focused on gene mutations in this study but did not rule out the potentials of other genomic changes that would similarly impact on RAS genes.

To the best of our knowledge, this is the first report of a comprehensive study focusing on RAS biology by examining high-throughput dependency and genomic datasets across such a diverse range of tumor types. Our findings shed light on the pivotal prominent pervasive role of RAS genes in oncogenesis and tissue-specific permissiveness of mutant K- or N-RAS oncogenesis. Collectively, our study provides compelling evidence from high-throughput CRISPR and genomics data, not only supporting the commonly-held critical oncogenic role of RAS genes, but also elevating our understanding of RAS biology by delineating in more refined knowledge and insights for the prominent pervasive oncogenic role and tissue-specific permissiveness of RAS gene mutation, which we hope to open new venues beyond current focus of RAS biology that warrant further investigation. This would benefit both clinical applications and basic research by promoting the awareness of RAS genes as the best candidates as oncogenic drivers amongst other oncogenes way beyond the current knowledge of RAS genes as the main driver gene for only 20-30% cancer types occurred in human beings clinical-wise or just the main focused tissue types including lung, colon, pancreas basic-research-wise. Practically, being aware of their prominent pervasive roles, we would be more concerned about the involvement of RAS genes in studying oncogenesis of tumors derived from a wider range of tissue types in wet lab research, and put RAS genes into priority of focus for treatment options in clinical setting. Furthermore, our results underscore the imperative for additional research endeavors aimed at elucidating the underlying molecular mechanisms and therapeutic ramifications of RAS genes in a myriad of cancer contexts.

The differential gene analysis of CRISPR screening data has also revealed significant insights into the potential other essential genes for RAS mutant and wild-type (WT) lines. Among the crucial findings, KRAS and NRAS, along with other oncogenic genes like RAF1 and SHOC2, were identified as potentially essential genes for RAS mutant lines. On the other hand, genes such as BRAF, SOS1, MAPK1, GRB2, and PTPN11 (i.e., SHP2) were found to be potentially essential for WT lines. SHOC2 forms a stable ternary signaling complex with MRAS and PP1C, leading to enhanced RAS-MAPK signaling in RASopathy Noonan syndrome^30^. Our study aligns with this observation, suggesting that SHOC2 plays a vital role in the survival advantage of RAS mutants in multiple tumor types (Skin, Haemato-lymphoid) by working with mutated RAS.

Many of the identified essential genes are also part of the common oncogenic RAS pathway annotated by the RAS Initiative^25^. Notably, they not only interact with RAS genes but also collaborate with each other in various cellular and genetic contexts. A notable example is PTPN11 (SHP2), which we found to be essential for WT lines from both lung and lymph/blood origin. SHP2 is a non-receptor protein-tyrosine phosphatase downstream of almost all RTKs, necessary for RTK-evoked RAS activation^31^. SHP2 inhibitors have demonstrated effectiveness in cancer models bearing RAS-GTP-dependent oncogenic BRAF, indicating its potential as a therapeutic target^32^. Furthermore, SHP2/MEK inhibitor combinations have shown promise in preventing adaptive resistance in various cancer models expressing mutant and wild-type KRAS^33^. These studies support the findings of our study in that SHP2, along with BRAF and SOS1, may work with or complement RAS gene in wild-type cell lines for oncogenesis, as they were identified as essential genes in our differential gene analysis of CRISPR effect score data.

The exhaustive computational screenings, involving large combinatorial trials of validation tests, served two essential purposes in our study. Firstly, they provided strong confirmation that the observed results from the differential analysis of CRISPR screening data between RAS mutant and WT lines were not occurring by random chances, thus solidifying the significant impact of RAS gene mutations. Secondly, these comprehensive screenings unexpectedly revealed many potential oncogenic driver genes, albeit to a lesser extent compared to RAS genes, but in a more tissue-specific manner. Among the identified genes were ALK, BRAF, PIK3CA, PIK3R1 (components of PI3 kinase), and CTNNB1, which are well-known oncogenic driver genes.

Furthermore, the computational screening results also shed light on seemingly less commonly known genes, such as WRN, which appeared in both independent sets of unique trials and multiple times in certain tissue types like colon, ovary, and stomach. Intriguingly, a smaller-scale CRISPR screening dataset al.so identified a biomarker-type dependency on WRN in colorectal and ovarian cell lines with MSI, suggesting its potential as a new synthetic lethal target in MSI tumors^34,35^. Moreover, WRN is critical for Werner syndrome and is associated with various mutations in cancer types such as peritoneal, colon, and stomach cancer^36^. The fact that the top differential genes derived from our exhaustive computational screenings were enriched with well-annotated oncogenic driver genes from prestigious computational studies on oncogenic driver genes^7–9^ further supports the oncogenic nature of these genes. Collectively, these observations suggest that the listed top differential genes derived from our analysis, like RAS genes and other well-known oncogenes, could potentially be putative oncogenic driver genes with previously unexplored biological relevance. Consequently, they merit further investigation to understand their presumed oncogenic roles and their cellular and genetic interactions with RAS genes.

Moreover, we initially observed a mutual exclusion mutation pattern between RAS genes and BRAF in various tumor types, such as Pancreas and Colon^26,27^, which is consistent with findings from the RAS Initiative’s RAS Dialogue^37^. Our study further strengthens this observation by identifying BRAF as a top gene with mutual exclusive mutation pattern with RAS genes in skin and colon cell lines from the DepMap database. This reinforces the essential role of BRAF in RAS wild type skin cell lines as identified through the differential gene analysis of CRISPR effect scores.

Our study unveiled that while tissue types naturally primed for KRAS or NRAS engagement tend to foster respective mutations, acquired KRAS or NRAS mutations take precedence over the initial tissue predisposition. These observations underscore the potent oncogenic influence of RAS gene mutations from an alternative perspective. A previous study demonstrated that distinct tissue-specific co-mutation networks are associated with each KRAS allele, leading to tissue-specific genetic dependencies linked to specific mutant KRAS alleles^4^. The impact of tissue-specificity on the oncogenic capacity of KRAS, concerning tissue permissiveness, was extensively discussed in a recent RAS Dialogue by the RAS Initiative^38^. One noteworthy finding from the authors of this RAS Dialogue is the observation that the KRAS tissue permissiveness pattern observed in mice closely resembles the cancer-type specific KRAS mutation frequency observed in patients. However, our current study pointed out that in those KRAS non-permissive tissue types, NRAS may be more likely to act as the oncogenic driver if conditions allowed. This inference is drawn from our discovery of KRAS- and NRAS-engaged tissue types, where the top differential genes in CRISPR effect scores between RAS mutant vs. WT and between NRAS vs. WT lines predominantly were revealed as NRAS rather than KRAS in a subset of NRAS-engaged tissue types (Fig. 1 and Supplementary Fig. 7). Our denoted NRAS-engaged tissue types seem coincident with many of the transformation-failed tissue-types that were mentioned in the RAS Dialogue article^38^ and cited references. Although the behaviors of converted lines observed in our study suggested that RAS gene mutations would be sufficient for conversion of either KRAS or NRAS dependency in cell lines, whether RAS gene mutations alone would be sufficient for oncogenic permissive for histological changes in non-permissive tissue types of the animal experiments is still a question to be further investigated. Nevertheless, our findings underscore the intriguing necessity of incorporating mutant NRAS or even HRAS alongside KRAS in the experiments to explore the tissue permissiveness of mutant RAS oncogenesis in those non-permissive tissue types, which were originally tested with KRAS alone.

Taken together, we proposed the concept of tissue-specific permissiveness of RAS gene mutations based on findings from DepMap data analysis in our study that were further inspired by the observed coincidence between KRAS or NRAS-engaged tissue types as identified from our study and the tissue-specific responses for their permissiveness to be transformed discussed in the RAS Dialogue^38^. Although we currently lack an in-depth mechanistic explanation or comprehensive understanding of the newly proposed tissue-specific permissiveness of RAS gene mutations, its implications for the long-standing puzzle that this RAS Dialogue discussed have inspired us to invite the community to undertake the unprecedented experimental efforts that would potentially find a way to transform the KRAS non-permissive tissue types that may help uncover the underlying oncogenic mechanisms for tissue-specific permissiveness and transformation capacity of mutated RAS genes.

While revealing the potential tissue-specific permissiveness, some converted cell lines were uncovered that can change the gene dependency behaviors of their RAS genes as opposed to their expected KRAS- or NRAS-engaged tissue types. Some of them appeared to resemble NRAS-dependent lines from NRAS-engaged tissue types although originally from KRAS-engaged tissue types presumably due to their acquired NRAS mutations, whereas others appear to resemble KRAS-dependent lines although originally from NRAS-engaged tissue types presumably due to their acquired KRAS mutations. Such “converted” changes may be attributed to the involvement of lineage plasticity or lost lineage fidelity as studied by others^39^. However, the initial analysis of transcriptome or proteome data from this study did not show obvious signs of lineage plasticity or lost lineage fidelity. One possibility is that the transcriptome or proteome data of DepMap we used are from original CCLE cell lines, but not those with loss of genes such as SMARCB1 related to lineage factor independence as described in another study^39^. As a result, we can not directly evaluate the lineage factor-related aspects on transcriptome and proteome of current DepMap data as in specifically designed experiments performed in the other study^39^. In addition, one study^1^ from our colleagues in the RAS program did show there are such RAS “converted” tumors in humans. A more detailed study on these converted lines is ongoing but beyond the scope of this study.

Moreover, our earlier research revealed that RAS gene expression is influenced by their mutational status, as well as by upstream and downstream genes^40^. Building on these insights, our current study utilized genomic data, including mutation status, gene expression from the same set of cell lines, providing robust support and consistency for the findings derived from both the differential gene analysis of CRISPR effect scores and the exhaustive computational screening analysis. Collectively, our observations strongly support the novel findings that RAS genes would act as the prominent pervasive oncogenic drivers across a wide range of tissue types. Oncogenic driver genes may interact with various other oncogenic driver genes that differ based on tissue origin, potentially acting as partners with RAS genes upon RAS mutation or even compensating for RAS genes in RAS wild type contexts, all of which contribute to oncogenesis. Our study thus further solidifies the pivotal role of RAS genes in cancer development and highlights their intricate interplay with other oncogenic drivers in a tissue-specific manner.

## Supplementary Information

Supplementary Material 1.  A supplementary information file accompanies this paper including Supplementary Fig. 1 to 18, and Supplementary Tables 1 to 7.

## Data Availability

The datasets generated during and/or analyzed during the current study are available from the corresponding author on reasonable request.
