# Supplementary material for: The prominent pervasive oncogenic role and tissue specific permissiveness of RAS gene mutations
Source: Sci Rep. 2024 Oct 26;14:25452. doi: 10.1038/s41598-024-76591-8 (PMC11511894; doi:10.1038/s41598-024-76591-8)
Supplement: Supplementary file 1 — Supplementary Material 1. A supplementary information file accompanies this paper including Supplementary Fig. 1 to 18, and Supplementary Tables 1 to 7. [file 41598_2024_76591_MOESM1_ESM.docx]

**Supplementary Information File for:**

**The prominent pervasive oncogenic role and tissue specific permissiveness of RAS gene mutations**

Ming Yi^1,^*, Daniel Soppet^1^, Frank McCormick^1,2^, Dwight V. Nissley^1^

^1^NCI RAS Initiative, Cancer Research Technology Program, Frederick National Laboratory for Cancer Research, Frederick, MD, USA.

^2^UCSF Helen Diller Family Comprehensive Cancer Center, San Francisco, CA, USA.

*Corresponding Author.

This document included Supplementary Figures 1 to 18 and Supplementary Tables 1 to 7


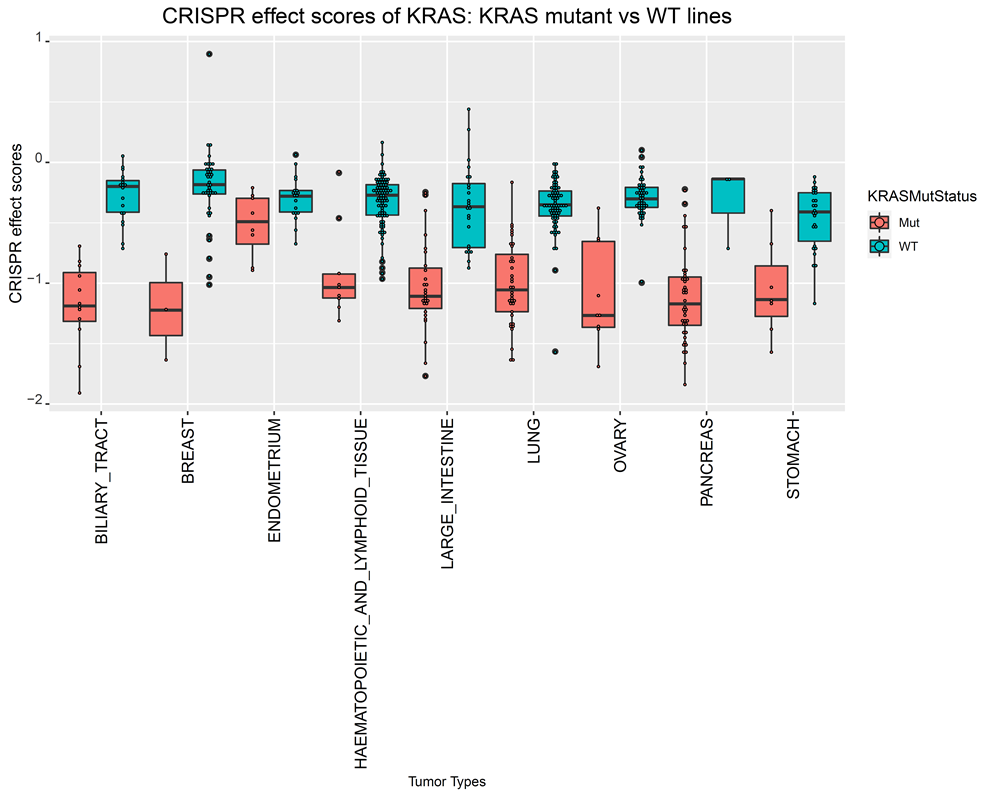


**Supplementary Figure 1**. Data mining of DepMap database showed difference of CRISPR effect scores of KRAS between KRAS mutant vs WT lines in many tumor types. Comparison of CRIPSR effect scores between KRAS mutant vs wild type (WT) lines from DepMap in boxplot. Only tumor types from DepMap with at least 3 samples in both KRAS mutant and WT lines would be used for comparison. Note: CRISPR effect scores of each gene reflects the dependency level on the corresponding gene in the cell line, where the more negative the value of the CRISPR effect score is, the more likely this cell line is more dependent on this gene.


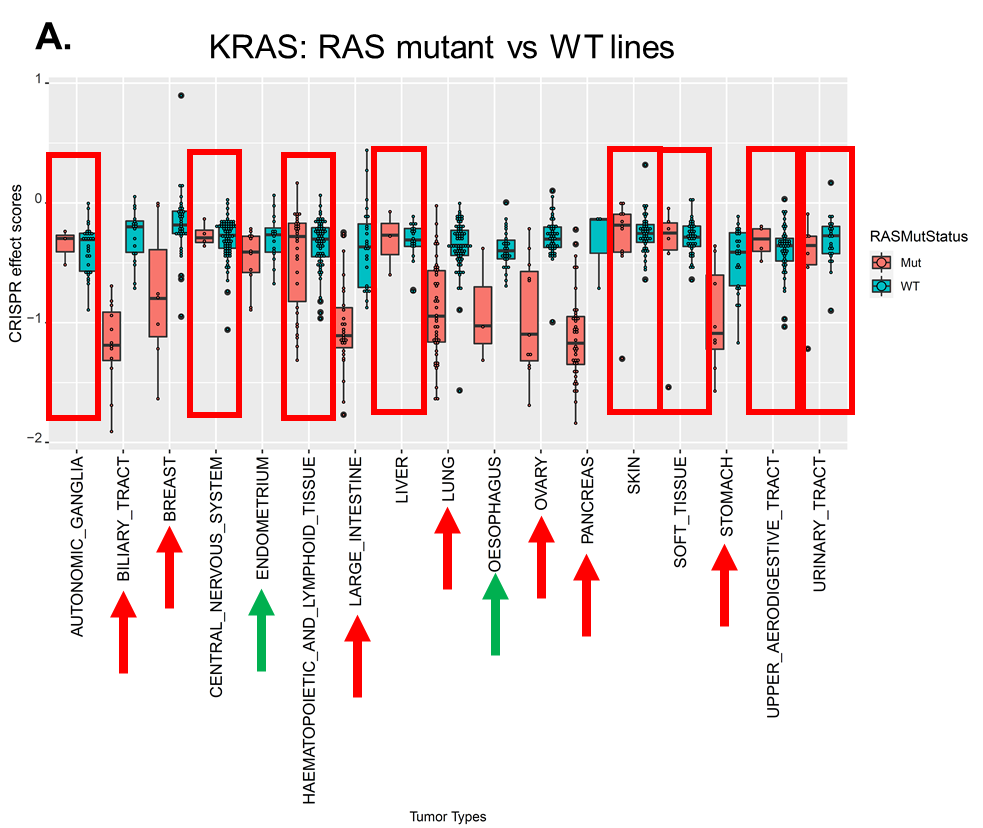


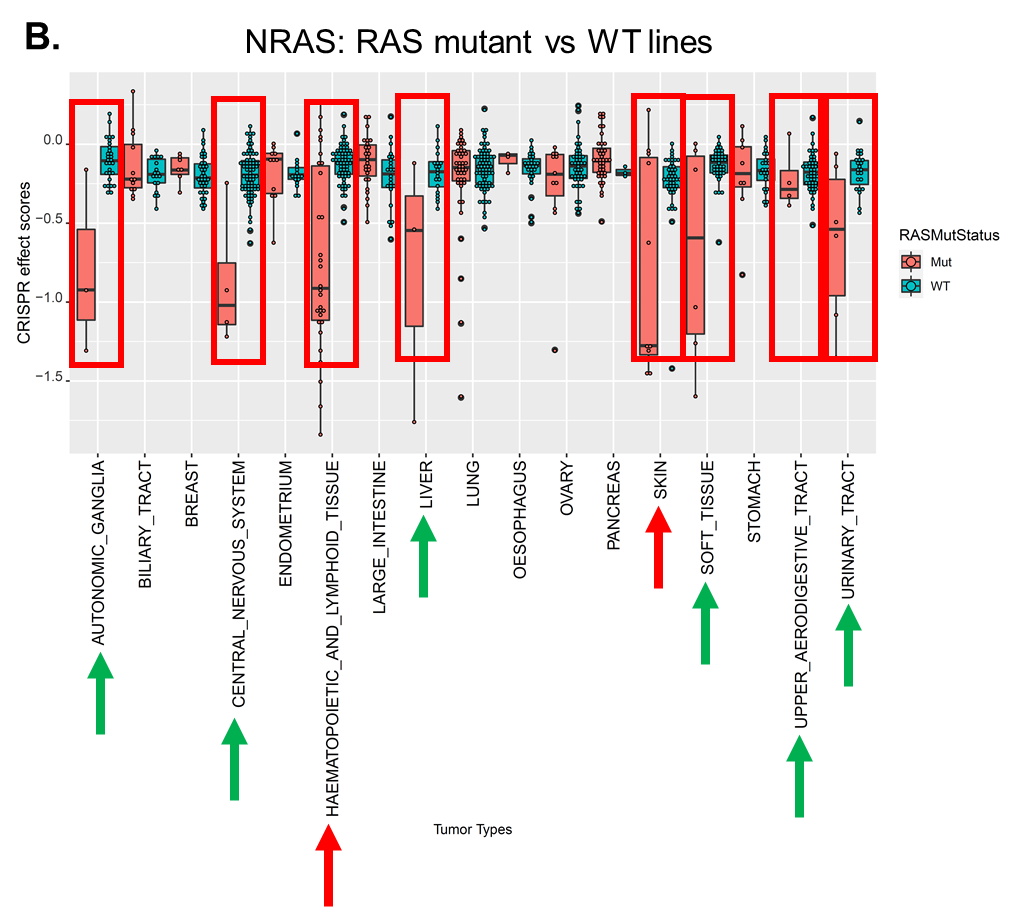


**Supplementary Figure 2.** Comparison of CRIPSR effect scores in boxplots of KRAS (left) or NRAS (right) between RAS mutant vs wild type (WT) lines from various tissue types in DepMap. Only tumor types from DepMap with at least 3 samples in both RAS (K-,N-,H-) mutant and WT lines would be used for comparison. **A.** KRAS gene’s CRIPSR effect scores were compared between RAS mutant vs WT lines in each tissue type with enough samples in each group (n>=3); **B.** NRAS gene’s CRIPSR effect scores were compared between RAS mutant vs WT lines in each tissue type with enough samples in each group (n>=3); Red arrows: native t-test p-value is significant; green arrows: native t-test p-value is not significant although there is a trend of difference in many cases. Red rectangles: highlight the tissue types where there is no difference or no negative shift of CRISPR effect scores for RAS mutant compared to WT lines for scores of KRAS, but there exists negative shift of CRISPR effect scores of NRAS for RAS mutant compared to WT lines. There are more tissue types shown here compared to **Supplementary Figure 1**, because inclusion of all Ras gene mutations added up more samples for comparison. Red arrow: native t-test p-value is significant; Green arrow: native t-test p-value is not significant. Note: CRISPR effect scores of each gene reflects the dependency level on the corresponding gene in the cell line, where the more negative the value of the CRISPR effect score is, the more likely this cell line is more dependent on this gene.


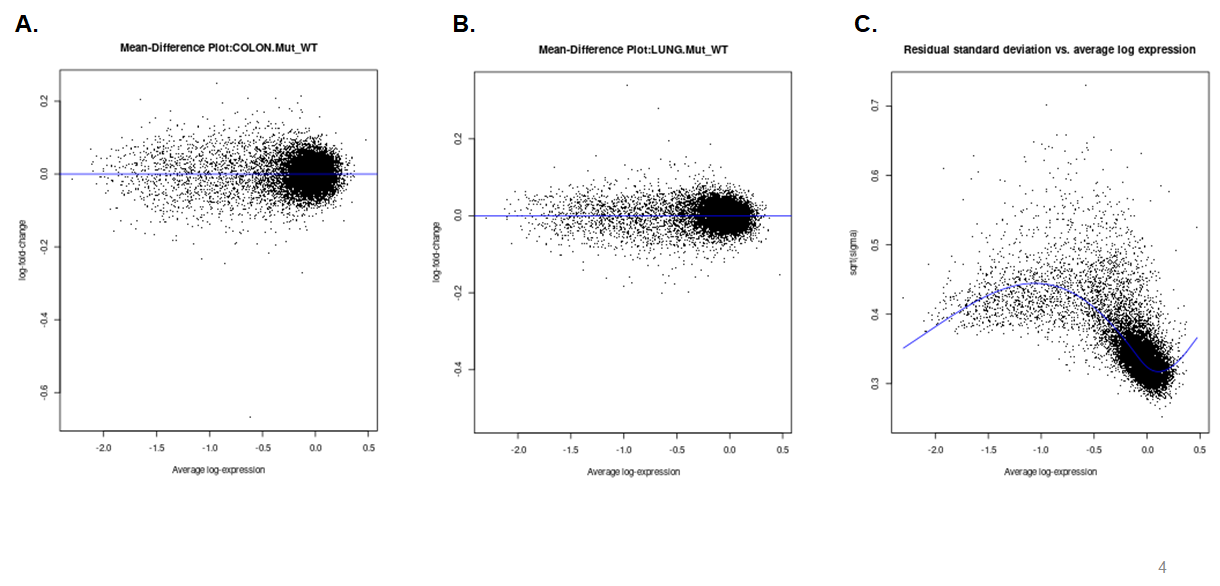


**Supplementary Figure 3.** Data assessment show a popular linear model-based method limma that is designed for high-throughput data and widely used for microarray data can be appropriately applied to the DepMap CRISPR gene effect score data. Limma method^20^ can obtain much higher significance levels than the native t-test for the same high-throughput datasets by improving statistical power through two ways: leverages information from the within-group replicates; borrows information across genes. These two approaches in combination increase the effective degrees of freedom dramatically. **A.** limma Mean-difference or MD plot of colon Mut vs WT lines; **B.** limma Mean-difference or MD plot of lung Mutant vs WT lines; **C.** limma SA plot (residual standard deviation vs. average log expression) for the fitted DepMap CRISPR effect score linear model. The y-axis is square-root fit$sigma, where sigma is the estimated residual standard deviation. The y-axis therefore corresponds to quarter-root variances.


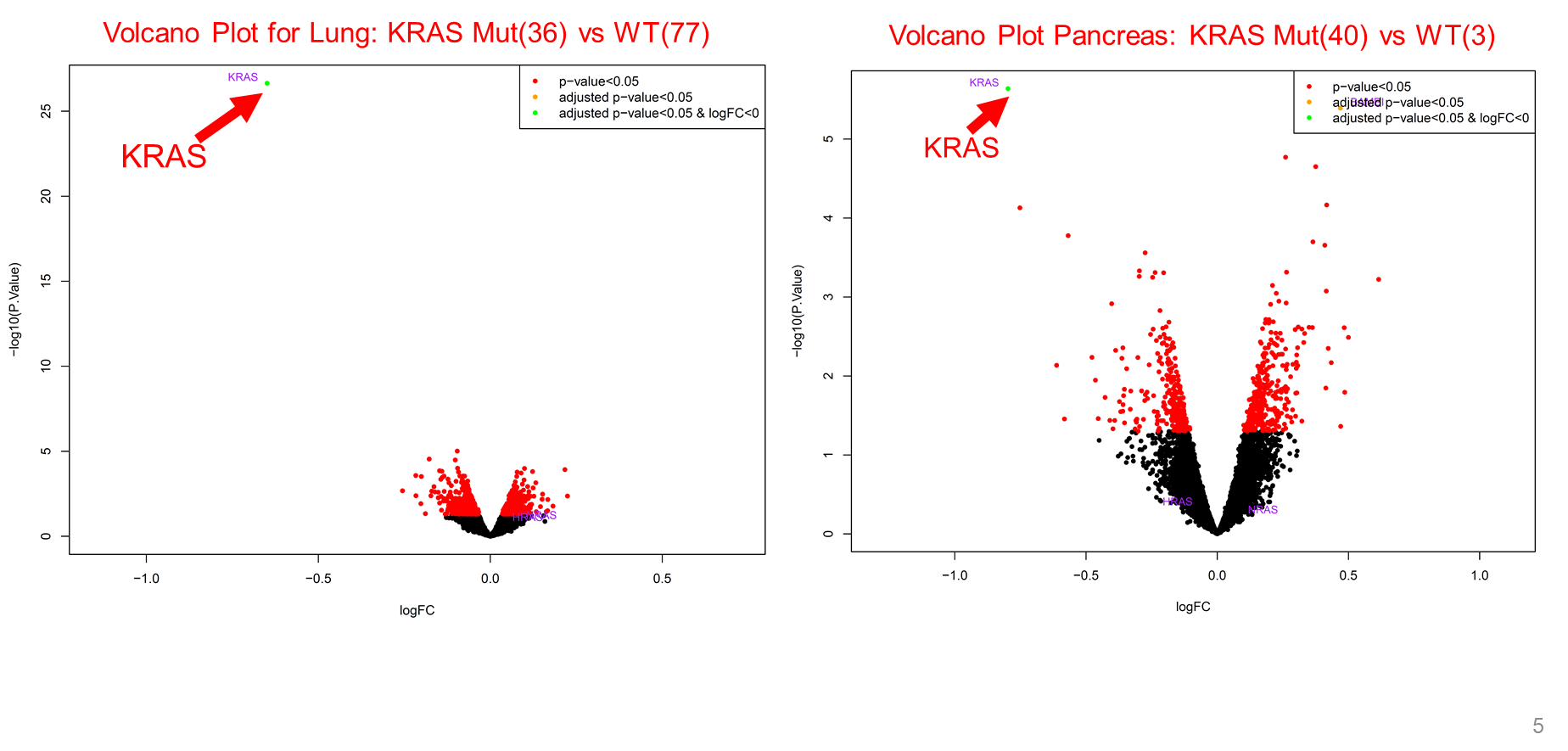
**Supplementary Figure 4.** KRAS is derived as the most significantly differential gene for CRISPR effect scores by multiple testing and with the most negative dependency difference between KRAS mutant vs WT lines from Lung and Pancreas. Volcano plots of all genes for CRISPR effect score data of DepMap between KRAS mutant vs WT lines from lung and pancreas tissue origin. **Left panel**: lung tissue origin; **Right panel**: pancreas tissue origin. Green data points: genes with significant adjusted p-value (<0.05) for multiple testing and logFC<0; orange data points: genes with significant adjusted p-value (<0.05) for multiple testing and logFC>=0; red data points: genes with significant raw p-value (<0.05); black data points: genes without statistical significance. The parentheses after “Mut” or “WT” indicate number of mutant lines or number of WT lines, respectively. Note: limma model is set up on the whole dataset including all tumor types, and so all data is under the same roof of the limma model, by which the power of the analysis was essentially increased as described earlier (Ritchie et al 2015, *Nucleic Acids Res*., 43(7):e47). X-axis logFC: log2 fold change as for the actual difference of the CRISPR effect scores between KRAS mutant vs WT lines in volcano plots, since the values of CRISPR effect scores inherently in logarithm transformed scale were used directly in limma; y-axis –log10(p.Value): (-1)*log10 of raw p-value of limma analysis.


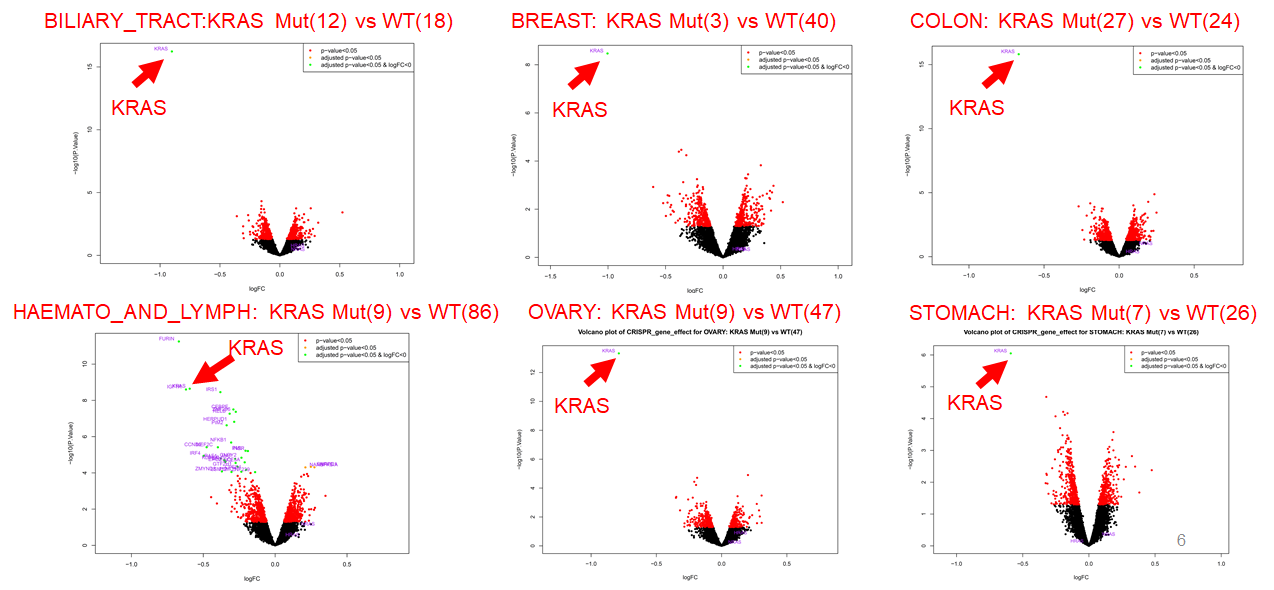


**Supplementary Figure 5.** KRAS is the most or nearly the most significantly differential gene for CRISPR effect scores by multiple testing and with the most or nearly the most negative dependency difference between KRAS mutant vs WT lines from various tissue types. Volcano plots of all genes for CRISPR effect score data of DepMap between KRAS mutant vs WT lines from various tissue origin. Green data points: genes with significant adjusted p-value (<0.05) for multiple testing and logFC<0; orange data points: genes with significant adjusted p-value (<0.05) for multiple testing and logFC>=0; red data points: genes with significant raw p-value (<0.05); black data points: genes without statistical significance. Note: limma model is set up on the whole dataset including all tumor types, and so all data is under the same roof of the limma model, by which the power of the analysis was essentially increased as described earlier^19^. The parentheses after “Mut” or “WT” indicate number of mutant lines or number of WT lines, respectively. X-axis logFC: log2 fold change as for the actual difference of the CRISPR effect scores between KRAS mutant vs WT lines in volcano plots, since the values of CRISPR effect scores inherently in logarithm transformed scale were used directly in limma; y-axis –log10(p.Value): (-1)*log10 of raw p-value of limma analysis. HAEMATO_AND_LYMPH: abbreviation for HAEMATOPOIETIC_AND_LYMPHOID_TISSUE. COLON: LARGE_INTESTINE.

**Supplementary Table 1.** Summary of limma results including top differential gene and top gene’s statistics between KRAS mutant vs WT lines from various tissue types from DepMap database as well as corresponding native t-test p-value for KRAS gene. Limma method improved the statistical power and uncovered the KRAS gene as the top differential gene in contrast of KRAS mutant vs WT lines across nearly all tissue types within high-throughput DepMap CRISPR datasets. Column “limma_TopGenes”: Top gene derived from limma analysis was listed for each tissue type contrast (KRAS mutant vs WT lines) if as KRAS gene or non-RAS gene; Column “limma_Statistics”: KRAS genes or other non-RAS gene (if KRAS gene is not Top gene) as Top 1, or 2 in each tissue type; adj.P.Val: Top genes were at level of adjusted P-value of multiple tests by limma; Column “adjusted.P-Val”: adjusted P-value of multiple tests by limma; Column “t-test_P-Values”: native t-test P-values. Note: tumor types in red with non-significant native t-test P-values but with significant adjusted p-value from limma method. Endometrium only detected GLUL gene in blue rather than KRAS as the top gene. HAEMATO_AND_LYMPH: abbreviation for HAEMATOPOIETIC_AND_LYMPHOID_TISSUE.


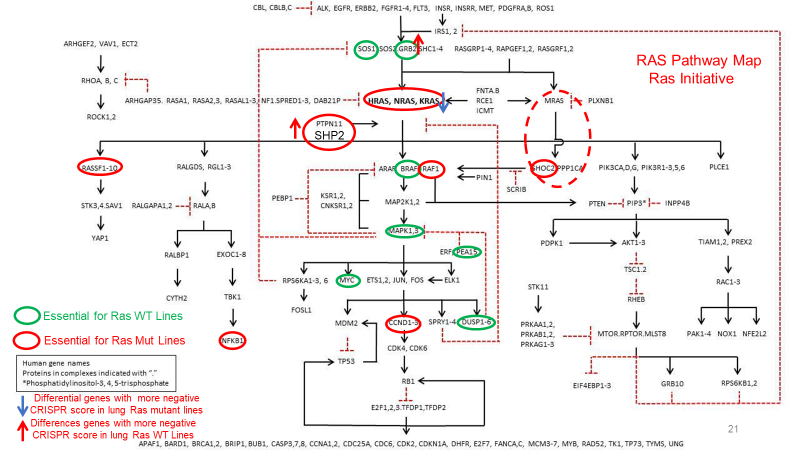


**Supplementary Figure 6.** Top differential genes of CRISPR effect scores between RAS mutant lines vs WT lines are essential genes for RAS mutants and wild type (WT) lines with significant underlying biological relevance with Ras biology. RAS pathway map of Ras Initiative with highlighted essential genes as derived differential genes with more negative CRIPSR effect score in lung RAS mutant lines (blue arrows) or in lung RAS wild type (WT) lines (red arrows). Ras pathway map is annotated by Ras Initiative at URL: https://www.cancer.gov/research/key-initiatives/ras/ras-central/blog/2015/ras-pathway-v2, which was also described in recent review^25^ from RAS Initiative. Note: Alias of PTPN11 is SHP2. Circled in colors are differential RAS pathway genes derived from contrasts of RAS mutant lines vs WT lines from limma method in many tissues with sufficient number of samples: genes in red circles: essential for RAS mutant lines; genes in green circles: essential for RAS WT lines (also see **Supplementary Table 2**). Dashed circle showed complex formed by the circled genes as supported by reference^30^ of Bonsor. *et al*.


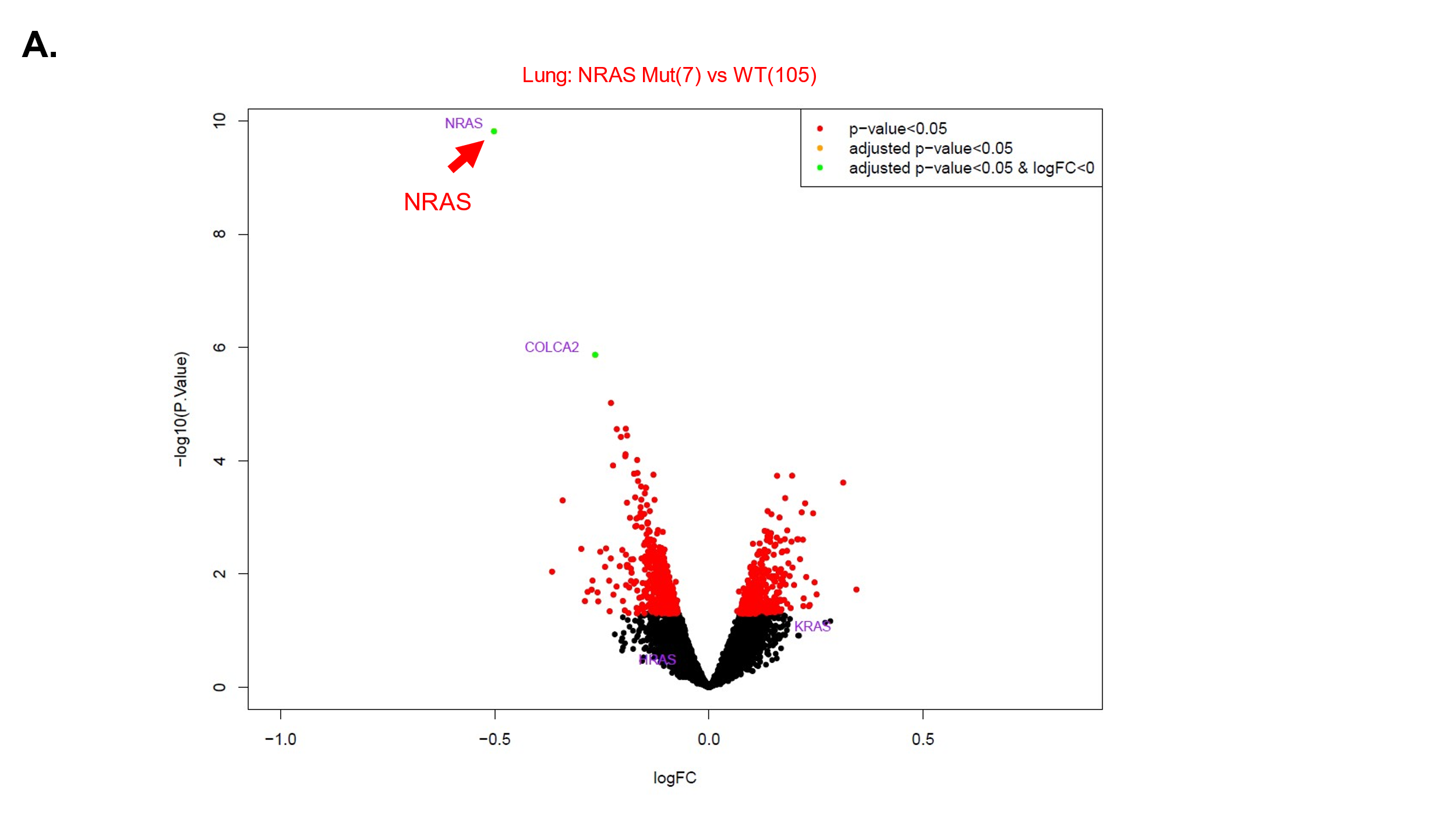


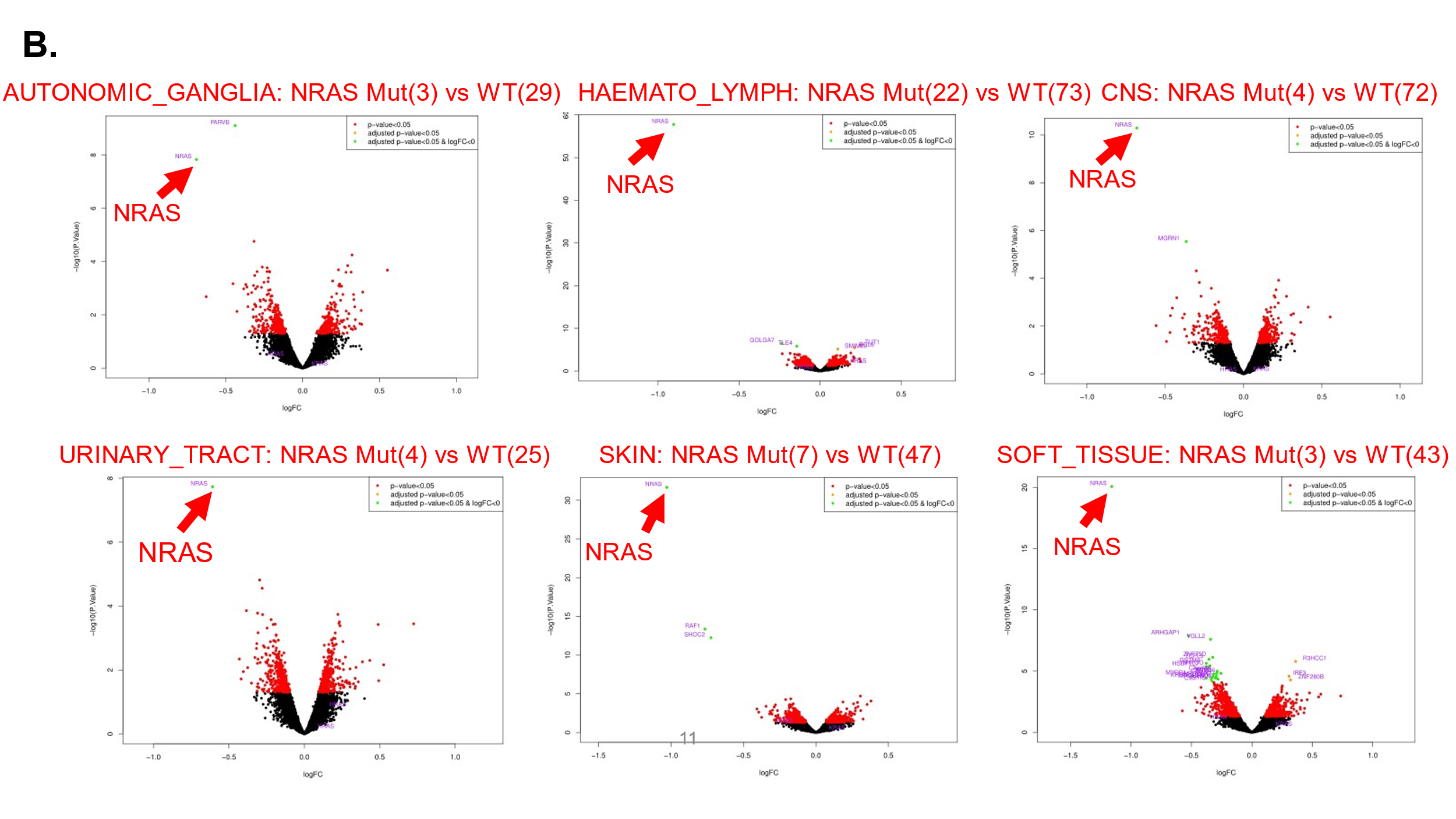


**Supplementary Figure 7.** NRAS is derived as the most or nearly the most significantly differential gene for CRISPR gene effect scores between NRAS mutant vs WT lines from subsets of tissue types. **A.** in Lung, NRAS was revealed as the top differential gene between NRAS mutant vs WT lines. . **B.** NRAS was revealed as the top differential gene between NRAS mutant vs WT in many tissue types. Green data points: genes with significant adjusted p-value (<0.05) for multiple testing and logFC<0; orange data points: genes with significant adjusted p-value (<0.05) for multiple testing and logFC>=0; red data points: genes with significant nominal p-value (<0.05); black data points: genes without statistical significance. The parentheses after “Mut” or “WT” indicate number of mutant lines or number of WT lines, respectively. Note: limma model is set up on the whole dataset including all tumor types, and so all data is under the same roof of the limma model, by which power of the analysis was essentially increased as described earlier^19^(Ritchie et al 2015, *Nucleic Acids Res*., 43(7):e47). X-axis logFC: log2 fold change as for the actual difference of the CRISPR effect scores between NRAS mutant vs WT lines in volcano plots, since the values of CRISPR effect scores inherently in logarithm transformed scale were used directly in limma; y-axis –log10(p.Value): (-1)*log10 of raw p-value of limma analysis. Colon: LARGE_INTESTINE; HAEMATO_LYMPH (abbreviation for

HAEMATOPOIETIC_AND_LYMPHOID_TISSUE); CNS: CENTRAL_NERVOUS_SYSTEM


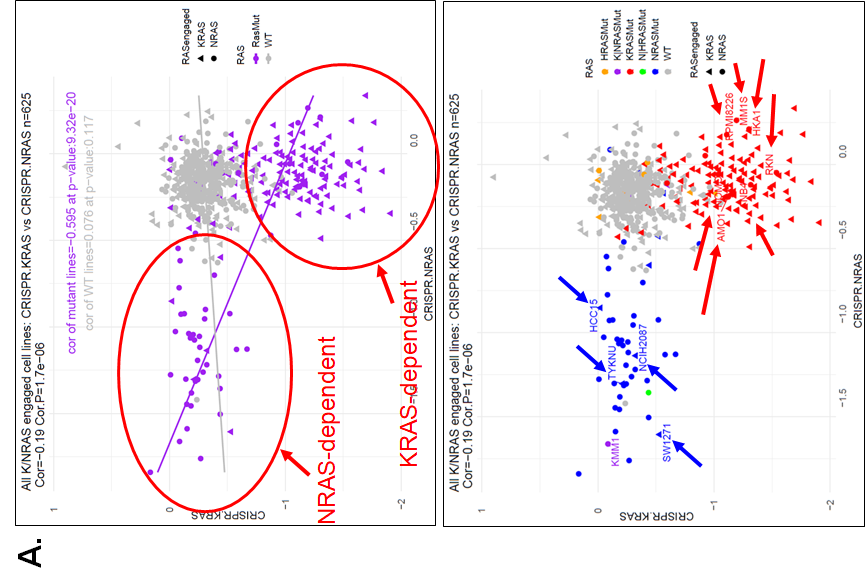


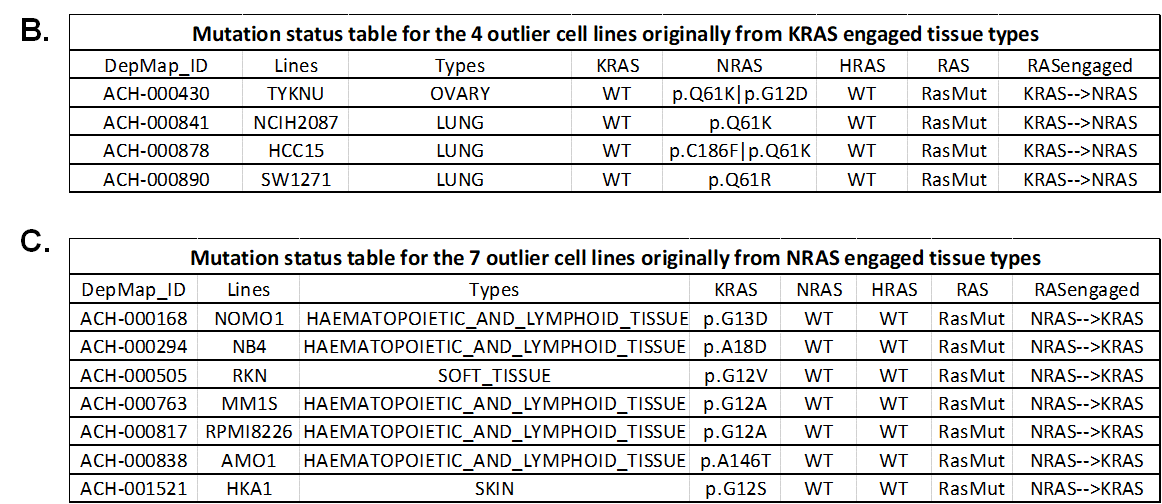


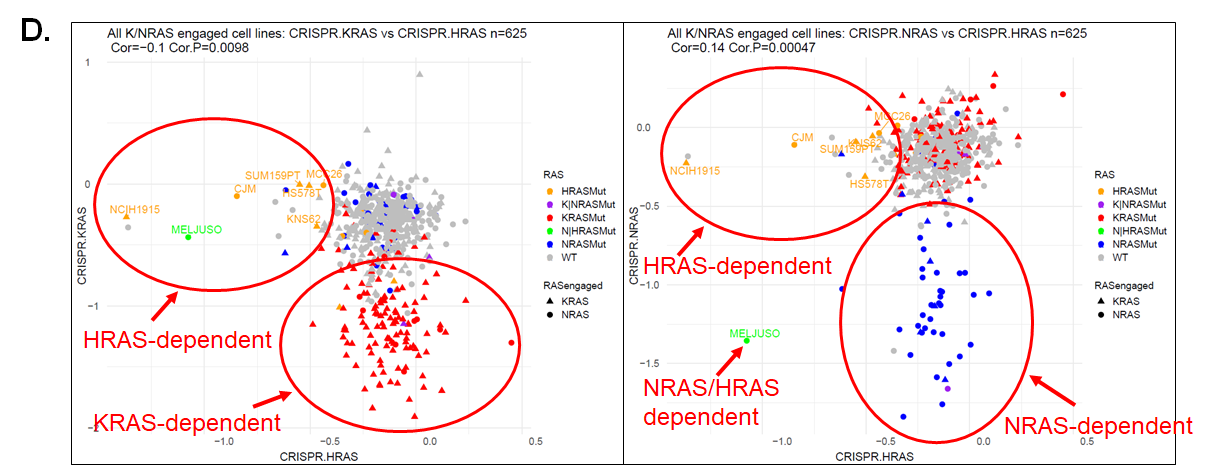


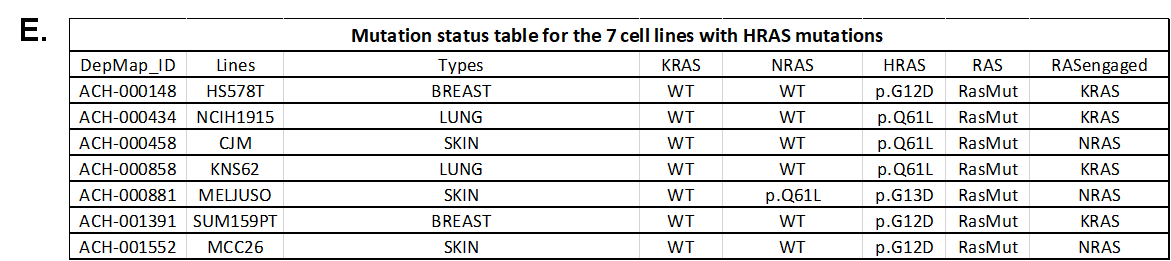


**Supplementary Figure 8.** (K- or N-) RAS gene mutations may determine the tissue-specific oncogenic capacity although preferentially cooperating with the revealed KRAS- or NRAS-engaged tissue types. The differential gene analysis of CRISPR effect score data between RAS mutant vs WT lines, between KRAS vs WT lines, or between NRAS vs WT lines (**Supplementary Figures 4,5,7** and **Figure** **1**) revealed the KRAS or NRAS as the most differential gene between corresponding mutant vs WT lines in subsets of tissue types, respectively, which allowed the denotation of KRAS-engaged tissue types: LUNG, PANCREAS, LARGE_INTESTINE, BREAST, OVARY, BILIARY_TRACT, STOMACH and NRAS-engaged tissue types: AUTONOMIC_GANGLIA, LIVER, CENTRAL_NERVOUS_SYSTEM, SKIN, SOFT_TISSUE, HAEMATOPOIETIC_AND_LYMPHOID_TISSUE, URINARY_TRACT. (note: in Lung, NRAS showed as the top differential gene between NRAS mutant vs WT lines, but KRAS as the top differential gene between RAS mutant vs WT lines, so tentatively assigned Lung as KRAS-engaged tissue type; in Liver, NRAS showed as top differential gene between RAS mutant vs WT lines, so tentatively assigned Liver as NRAS-engaged tissue type; in HAEMATOPOIETIC_AND_LYMPHOID_TISSUE, NRAS showed as top differential gene between RAS mutant vs WT lines, although KRAS and NRAS as top differential gene between KRAS vs WT or between NRAS vs WT respectively, tentatively assigned as NRAS-engaged tissue types). **A.** Scatter plots of CRIPSR effect scores of KRAS gene (CRISPR.KRAS at y-axis) vs CRIPSR effect scores of NRAS gene (CRISPR.NRAS at x-axis) for all cell lines from either KRAS-engaged or NRAS-engaged tissue types; **Top panel**: scatter plot of intended cell lines with regression lines for RAS mutant lines (in purple) and for RAS WT (wild type) lines (in gray) showing corresponding correlation coefficients and related p-values in plot; RAS mutants showed much better negative correlation (cor=-0.595) between CRIPSR effect scores of KRAS gene vs NRAS gene comparing to much lower correlation in all lines (cor=-0.19 for both RAS mutant and WT lines) or no correlation in WT lines (cor=0.076); RAS mutation status shown in purple and WT lines in gray. Cell lines from KRAS-engaged tissue types in triangles and from NRAS-engaged tissue types in circles, respectively; The negative CRISPR effect scores presumably put the corresponding cell lines as KRAS-, or NRAS-dependent as indicated by large red circles and red arrows; **Bottom panel**: same scatter plot except with more detailed mutation status for RAS genes and some converted lines indicated by arrows; cell lines indicated by blue arrows or red arrows are converted lines either clustered with NRAS mutant lines (in blue circles) although originally from KRAS engaged tissue types (in blue triangles) presumably due to their acquired NRAS mutations (see table in **B**), or clustered with KRAS mutant lines (in red triangles) although originally from NRAS-engaged tissue types (in red circles) presumably due to their acquired KRAS mutation (see table in **C**), respectively. **B.** The mutation status table showed that the 4 converted cell lines originally from KRAS-engaged tissue types (the cell lines as the blue triangles indicated by blue arrows in **Bottom panel** of **A**) all have NRAS mutations rather than KRAS mutations, which presumably may make them behave more like NRAS-engaged tissue types (clustered with the NRAS mutant lines in blue circles from NRAS-engaged tissue types; see **Bottom panel** of **A**); Columns: KRAS, NRAS, HRAS, RAS: mutation status of RAS genes (column RAS is combined status of all RAS genes); RASengaged: NRAS- or KRAS-engaged tissue types. KRAS🡪NRAS represents the conversion of original KRAS engaged tissue types into NRAS-engaged tissue types for the corresponding 4 converted lines presumably due to their acquired NRAS mutations shown in the table. **C.** The mutation status table showed that the 7 converted cell lines originally from NRAS-engaged tissue types (the cell lines as the red circles indicated by red arrows in **Bottom panel** of **A**) all have KRAS mutations rather than NRAS mutations, which may make them behave more like KRAS-engaged tissue types (clustered with the KRAS mutant lines in red triangles from KRAS-engaged tissue types, see **Bottom panel** of **A**); Columns: KRAS, NRAS, HRAS, RAS: mutation status of RAS genes (column RAS is combined status of all RAS genes); RASengaged: NRAS- or KRAS-engaged tissue types. NRAS🡪KRAS represents the conversion of original NRAS-engaged tissue types into KRAS-engaged tissue types for the corresponding 7 converted lines presumably due to their acquired KRAS mutations shown in the table. **D.** Scatter plots of CRIPSR effect scores of HRAS gene (CRISPR.HRAS at x-axis) vs CRIPSR effect scores of KRAS gene (CRISPR.KRAS at y-axis, **Left panel**) or vs CRIPSR effect scores of NRAS gene (CRISPR.NRAS at y-axis, **Right panel**) for all cell lines from either KRAS-engaged or NRAS-engaged tissue types. The negative CRISPR effect scores presumably put the corresponding cell lines as HRAS-, KRAS-, NRAS-, or NRAS/HRAS-dependent as indicated by large red circles and/or red arrows. **E.** The table showed that the mutation status of the 7 HRAS or HRAS/NRAS mutant cell lines (in orange or green; see panel **D**) may presumably make these cell lines HRAS-dependent or NRAS/HRAS-dependent.


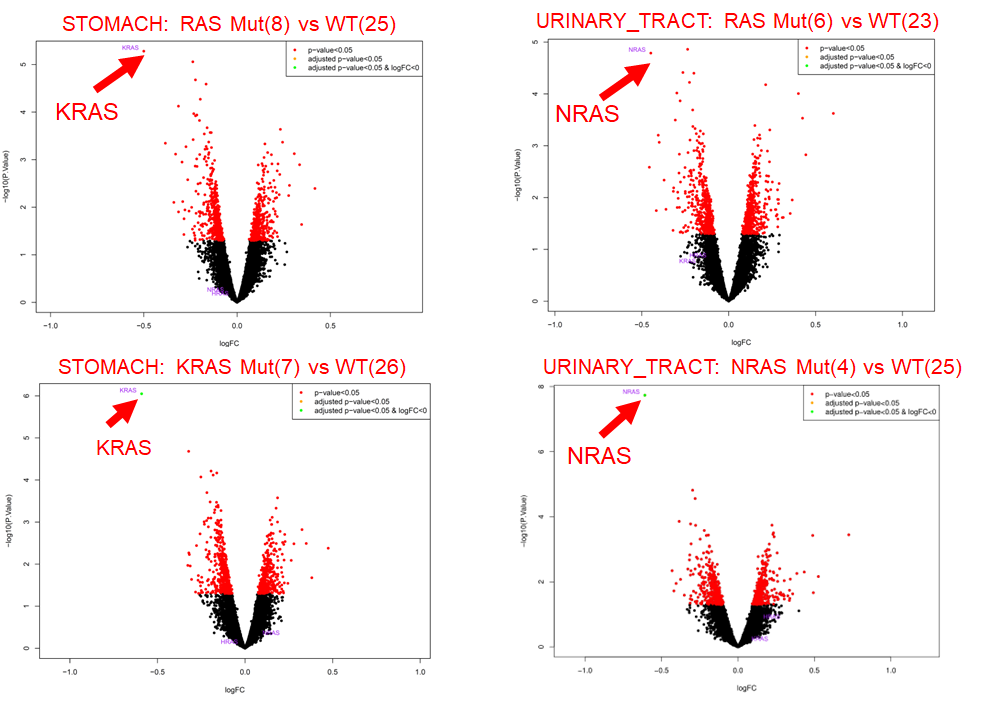


**Supplementary Figure 9.** KRAS or NRAS is derived as the most or nearly the most significantly differential gene for CRISPR gene effect at raw p-value<0.05 with the most or nearly most negative dependency difference between RAS mutant vs WT lines from stomach and urinary_tract tissue types. Volcano plots of all genes for CRISPR effect score data of DepMap between RAS mutant vs WT lines from stomach and urinary tract tissue types. Top-left panel: stomach tissue origin; Top-right panel: urinary_tract tissue origin for contrast of RAS mutant vs WT lines; Bottom-right panel: urinary_tract tissue origin for contrast of NRAS mutant vs WT lines. Green data points: genes with significant adjusted p-value (<0.05) for multiple testing and logFC<0; orange data points: genes with significant adjusted p-value (<0.05) for multiple testing and logFC>=0; red data points: genes with significant raw p-value (<0.05); black data points: genes without statistical significance. The parentheses after “Mut” or “WT” indicate number of mutant lines or number of WT lines, respectively. Note: limma model is set up on the whole dataset including all tumor types, and so all data is under the same roof of the limma model, by which the power of the analysis was essentially increased as described earlier (Ritchie et al 2015, *Nucleic Acids Res*., 43(7):e47). X-axis logFC: log2 fold change as for the actual difference of the CRISPR effect scores between RAS mutant vs WT lines in volcano plots, since the values of CRISPR effect scores inherently in logarithm transformed scale were used directly in limma; y-axis –log10(p.Value): (-1)*log10 of raw p-value of limma analysis. Note: In stomach and urinary_tract, contrast of RAS Mut vs WT did not give KRAS or NRAS with statistical significance of multiple tests for adjusted p-value, although still as the top gene with significant p-value respectively (top-left and top-right, respectively). But in urinary_tract, the contrast of NRAS mutant vs WT did give NRAS not only the top gene but also with the statistical significance of multiple tests with adjusted p-value <0.05 (bottom-right); the same story for stomach when comparing KRAS vs WT lines (bottom-left) (Also see **Supplementary Figure 5**).


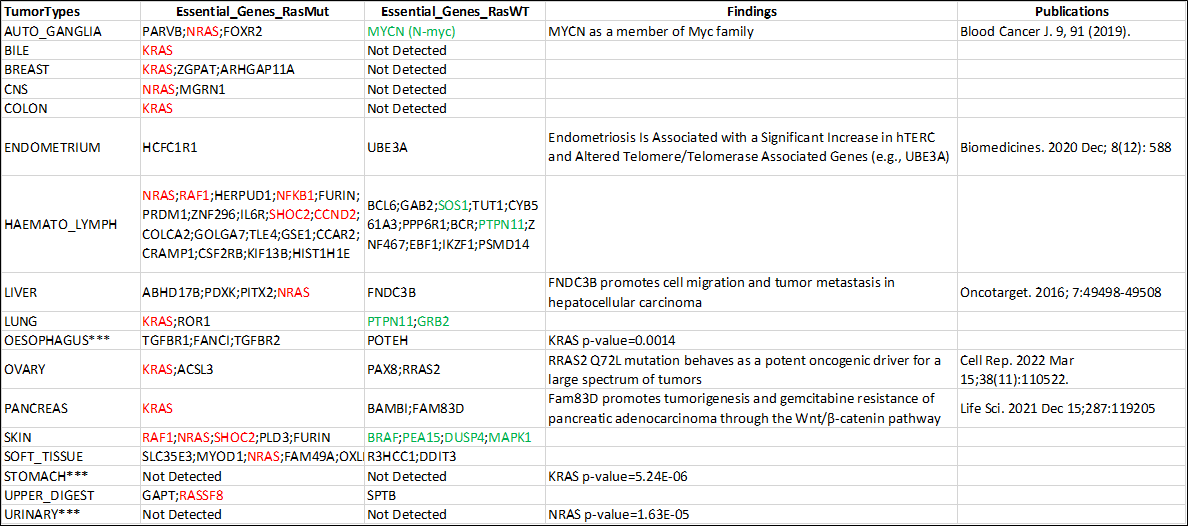


**Supplementary Table 2.** Summary of essential genes for RAS mutant lines and for RAS WT lines as derived as differential genes (adjusted p-value<0.05 by limma) in CRISPR effect score data from DepMap for various tumor types. Based on whether the differential genes have more negative scores in RAS mutant lines or in WT lines, they are classified as either potentially as essential genes for RAS Mutant lines (2^nd^ column), or as essential genes for WT lines (3^rd^ column). Note: colored genes (red as essential for RAS mutants, green for WT) are from RAS pathway (RAS program annotation). *******: Not detected at adjusted p-value <0.05 with RAS genes, but KRAS or NRAS as the top differential genes at raw p-value<0.05.


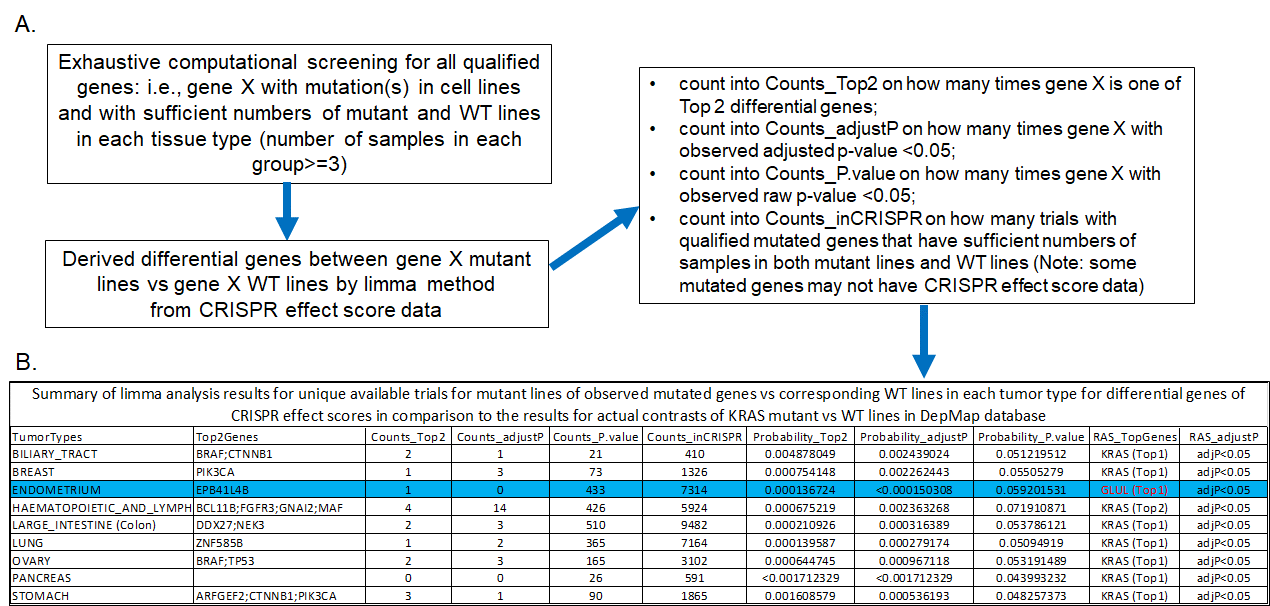


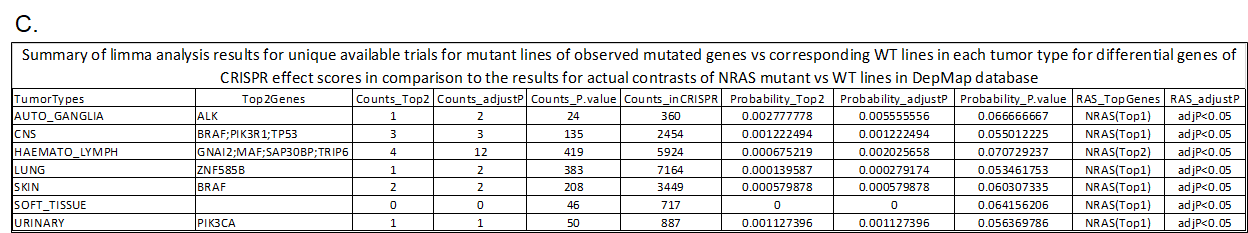


**Supplementary Figure 10.** Procedure and summarized results for exhaustive computational screenings of all observed mutated genes vs corresponding WT lines in each tumor type where KRAS was derived as the top differential genes of CRISPR effect scores between KRAS vs WT lines (**B**) or those for NRAS between NRAS vs WT lines (**C**) from DepMap database. **A**. The procedure of exhaustive computational screenings. **B**. The summarized results for exhaustive computational screenings of all observed mutated genes vs corresponding WT lines in each tumor type where KRAS was derived as the top differential genes of CRISPR effect scores between KRAS vs WT lines, which suggested that the observation of KRAS as the most significant differential gene of the CRISPR effect scores between KRAS mutant vs WT lines (see **Supplementary Figure 4 and 5)** in multiple tissue types (KRAS-engaged tissue types) are not possibly occurring by random chances. The 2^nd^ column of the summary table (Column Top2Genes) listed mutated genes in these screenings which behaved like KRAS in that comparison of the corresponding mutant lines vs. WT lines for these genes did derive themselves as the top (or top 2) differential genes by the limma method for each corresponding tissue type observed from the exhaustive computational screenings. The procedure outlined above the table also showed how columns 3 to 6 have been collated from screening data. Column Probability_Top2 was derived as division of Counts_Top2 in column 3 by Counts_inCRISPR in column 6; column Probability_adjustedP was derived as division of Counts_adjustP in column 4 by Counts_inCRISPR in column 6; column Probability_P_value was derived as division of Counts_P_value in column 5 by Counts_inCRISPR in column 6; column RAS_TopGenes list the top (or top 2) differential gene derived mainly as KRAS in the comparison of KRAS mutant vs WT lines by limma method. Last column listed the corresponding statistically significant level for column RAS_TopGenes. Blue row indicated that the endometrium is the only one originally failed to derive KRAS gene as the top differential gene (see **Supplementary Table 1**). **C**. The summarized results for exhaustive computational screenings of all observed mutated genes vs corresponding WT lines in each corresponding tumor type where NRAS was derived as the top differential genes of CRISPR effect scores between NRAS vs WT lines, which also suggested similar conclusion that NRAS as the most significant differential gene of the CRISPR effect scores between NRAS mutant vs WT lines (see **Supplementary Figure 7)** in those corresponding tissue types (NRAS-engaged tissue types) are not possibly occurring by random chances. HAEMATO_LYMPH: abbreviation for HAEMATOPOIETIC_AND_LYMPHOID_TISSUE; CNS: CENTRAL_NERVOUS_SYSTEM. URINARY: URINARY_TRACT


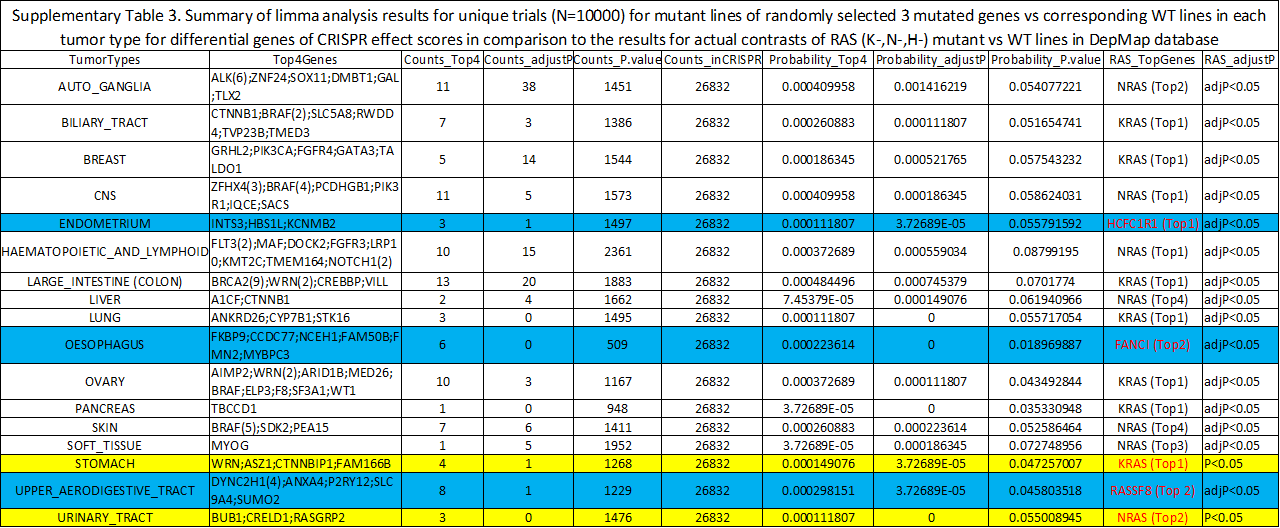


**Supplementary Table 3.** The summarized results in the table suggested that the observation of RAS genes as the most significant differential gene of the CRISPR effect scores between RAS mutant vs WT lines in multiple tumor types are not possibly occurring by random chances. Computational screening of unique trials (n=10000) for randomly selected 3 mutated genes vs corresponding WT lines in each tumor type for differential genes of CRISPR effect scores were performed to validate that the observation of RAS gene(s) as the top significant differential genes of the CRISPR effect scores between RAS mutant vs WT lines in multiple tumor types are not possibly occurring by random chances. The 2^nd^ column of the summary table (Column Top4Genes) listed mutated genes in these screenings which behaved like RAS genes in that comparison of the corresponding mutant lines vs. WT lines for these genes did derive themselves as the top (or top 4) differential genes by the limma method for each corresponding tissue type observed from the exhaustive computational screenings. The similar procedure has been used to collate columns 3 to 6 from screening data. Column Probability_Top 4 was derived as division of Counts_Top4 in column 3 by Counts_inCRISPR in column 6; column Probability_adjustedP was derived as division of Counts_adjustP in column 4 by Counts_inCRIPR in column 6; column Probability_P_value was derived as division of Counts_P_value in column 5 by Counts_inCRISPR in column 6; column RAS_TopGenes list the top (or top 4) differential gene derived mainly as RAS genes in the comparison of RAS mutant vs WT lines by limma method. Last column lists the corresponding statistically significant level for column RAS_TopGenes. The numbers in parentheses indicate how many times the corresponding genes were observed as the Top 4 genes in the unique trials. Light blue rows are tissue types without RAS genes derived as top 4 genes in Ras mutant vs WT contrasts. Yellow rows are tissue types with RAS genes derived as top 4 genes but at level of raw p-value (<0.05).

**Supplementary Table 4.** The summarized results in the table suggested that the observation of RAS genes as the most significant differential gene of the CRISPR effect scores between RAS mutant vs WT lines in multiple tumor types are not possibly occurring by random chances. Computational screening of another set of unique trials (n=10000) for randomly selected 3 mutated genes vs corresponding WT lines in each tumor type for differential genes of CRISPR effect scores were performed to validate that the observation of RAS gene(s) as the top significant differential genes of the CRISPR effect scores between RAS mutant vs WT lines in multiple tumor types are not possibly occurring by random chances. The 2^nd^ column of the summary table (Column Top4Genes) listed mutated genes in these screenings which behaved like RAS genes in that comparison of the corresponding mutant lines vs. WT lines for these genes did derive themselves as the top (or top 4) differential genes by the limma method for each corresponding tissue type observed from the exhaustive computational screenings. The similar procedure has been used to collate columns 3 to 6 from screening data. Column Probability_Top4 was derived as division of Counts_Top4 in column 3 by Counts_inCRISPR in column 6; column Probability_adjustedP was derived as division of Counts_adjustP in column 4 by Counts_inCRISPR in column 6; column Probability_P_value was derived as division of Counts_P_value in column 5 by Counts_inCRISPR in column 6; column RAS_TopGenes list the top (or top 4) differential gene derived mainly as RAS genes in the comparison of RAS mutant vs WT lines by limma method. Last column lists the corresponding statistically significant level for column RAS_TopGenes. The numbers in parentheses indicate how many times the corresponding genes were observed as the Top 4 genes in the unique trials. Light blue rows are tissue types without RAS genes derived as top 4 genes in Ras mutant vs WT contrasts. Yellow rows are tissue types with RAS genes derived as top 4 genes but at level of raw p-value (<0.05).

**Supplementary Table 5.** The oncogenic driver genes (defined by tumor types) were enriched within differential genes (which are also one of the starting randomly selected 3 mutated genes) of CRISPR effect scores between the corresponding mutant vs WT lines of unique trials (n=10000) in many tumor types. Here only use the annotation of the oncogenic driver genes defined by tumor types in the corresponding studies, which are collated from an ICGC PCAWG-based study^8^ and two TCGA based studies^7,9^. Column Num_DEGs: number of unique DEGs (at adjusted p-val<0.05), which are also one of the starting randomly selected 3 mutated genes; column Enrichment_P.value: enrichment p-values derived from corresponding Fisher’s exact tests; column DEGs_List: unique DEGs for each tissue type with oncogenic driver genes in bold, which were annotated by prestigious computational studies on oncogenic driver genes^7,8,9^; green rows: Num_DEGs>=2 and with significant enrichment of well-annotated driver genes; red rows: Num_DEGs>=2 and without significant enrichment of well-annotated driver genes; yellow rows: Num_DEGs=1; blue rows: Num_DEGs=0. Enrichment analysis is evaluated by Fisher's exact test on a typical 2X2 contingency table created for each tissue type on status of a DEG (differential CRISPR effect score gene) (whether the gene is driver gene or not versus whether it is a DEG or not in each tissue type) within these unique trials of the computational screenings for each tissue type.

**Supplementary Table 6.** The oncogenic driver genes (not defined by tumor types) were enriched within differential genes (which are also one of the starting randomly selected 3 mutated genes) of CRISPR effect scores between the corresponding mutants vs WT lines of unique trials (n=10000) in many tumor types. Driver genes in bold in column DEGs_List. Here only use the annotation of the oncogenic driver genes not defined by tumor types in the corresponding studies, which are collated from an ICGC PCAWG-based study^8^ and two TCGA based studies^7,9^. Column Num_DEGs: number of unique DEGs (at adjusted p-val<0.05), which are also one of the starting randomly selected 3 mutated genes; column Enrichment_P.value: enrichment p-values derived from corresponding Fisher’s exact tests; column DEGs_List: unique DEGs for each tissue type with oncogenic driver genes in bold, which were annotated by prestigious computational studies on oncogenic driver genes^7,8,9^; green rows: Num_DEGs>=2 and with significant enrichment of well-annotated driver genes; red rows: Num_DEGs>=2 and without significant enrichment of well-annotated driver genes; yellow rows: Num_DEGs=1; blue rows: Num_DEGs=0. Enrichment analysis is evaluated by Fisher's exact test on a typical 2X2 contingency table created for each tissue type on status of a DEG (differential CRISPR effect score gene) (whether the gene is driver gene or not versus whether it is a DEG or not in each tissue type) within these unique trials of the computational screenings for each tissue type.

**Supplementary Table 7.** Top genes with the most significant association between presence of mutations in the corresponding gene and the dependency of this gene within all cell lines with mutation and CRISPR screening data are mainly oncogenic driver genes. Table of the top genes out of ~17k genes with the most significant association between presence of mutations in a gene and the dependency on this corresponding gene denoted by its CRISPR effect score <(-1.0) (a score of -1 corresponds to the median of all common essential genes) across all cell lines of all tissue types in DepMap database. Significant association between the presence of mutations in the corresponding gene and the dependency of this gene within all the qualified DepMap CCLE cell lines were evaluated by Fisher's exact test with multiple-testing correction by Benjamini-Hochberg method and ranked by the enrichment p-values. Enrichment.p.Val: raw enrichment p-value was derived by Fisher’s exact test on a typical 2X2 contingency table created for all cell lines on status of a gene (whether the gene has mutation(s) or not versus whether it has a CRISPR effect scores <(-1.0) or not in each cell line); Enrichment.Adjusted.p.Val: adjusted Enrichment.p.Val by Benjamini-Hochberg method; Wilcox.p.val: p-value derived from Wilcoxon tests; Wilcox.Adjusted.p.Val: adjusted Wilcox.p.val by Benjamini-Hochberg method; t.test.p.val: p-value derived from t-test tests; t.test.Asjusted.p.Val: adjusted t.test.p.val by Benjamini-Hochberg method; mean.Diff: mean difference between the means of mutant lines vs WT lines; Odd_ratio: Odd_ratio from Fisher’s exact test (one-sided for enrichment); Mut_Dep_Lines: how many cell lines with the corresponding gene mutated and having CRISPR effect score <(-1.0); Mut_NotDep_Lines: how many lines with the corresponding gene mutated and but not having CRISPR effect score <(-1.0); NotMut_Dep_Lines: how many lines without the corresponding gene mutated but having CRISPR effect score <(-1.0); TotalLines: total number of qualified DepMap CCLE cell lines with both mutation status and CRISPR effect score data; DriverGenes: whether the gene is a oncogenic driver gene based on references (see note behind). Note: Only genes in green background are at significance of Adjusted p.value <0.05. Total 945 cell lines in multiple tissue/tumor types with both mutation and DepMap CRISPR effect score data available have been tested for every of total 16925 genes and showed here are only the top ranked list. Oncogenic driver gene annotations are collated from an ICGC PCAWG-based study^8^ and two TCGA based studies^7,9^.


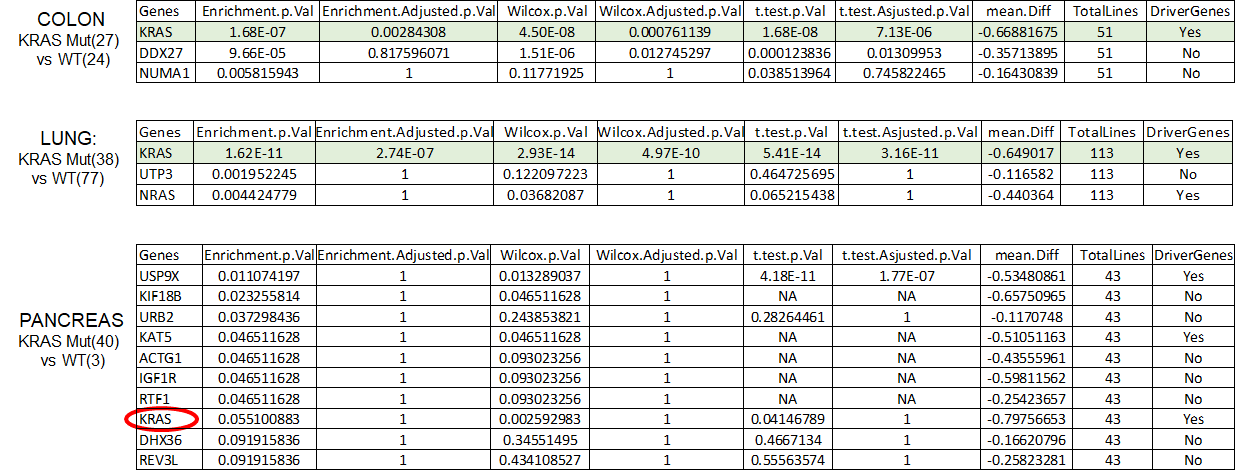


**Supplementary Figure 11.** KRAS as the top gene with the most significant association between presence of mutations in the corresponding gene and the dependency of this gene in colon and lung. Tables of the top genes out of ~17k genes with the most significant association between presence of mutations in a gene and the dependency on this corresponding gene denoted by its CRISPR effect score <(-1.0) (a score of -1 corresponds to the median of all common essential genes) across all cell lines of colon or lung although not in pancreas (likely impacted by limited sample size of WT lines). Significant association between the presence of mutations in the corresponding gene and the dependency of this gene within all the qualified DepMap CCLE cell lines were evaluated by Fisher's exact test with multiple-testing correction by Benjamini-Hochberg method and ranked by the enrichment p-values. Enrichment.p.Val: raw enrichment p-value was derived by Fisher’s exact test on a typical 2X2 contingency table created for all cell lines of each tissue type on status of a gene (whether the gene has mutation(s) or not versus whether it has a CRISPR effect scores <(-1.0) or not in each cell line); Enrichment.Adjusted.p.Val: adjusted Enrichment.p.Val by Benjamini-Hochberg method; Wilcox.p.val: p-value derived from Wilcoxon tests; Wilcox.Adjusted.p.Val: adjusted Wilcox.p.val by Benjamini-Hochberg method; t.test.p.val: p-value derived from t-test tests; t.test.Asjusted.p.Val: adjusted t.test.p.val by Benjamini-Hochberg method; mean.Diff: mean difference between the means of mutant lines vs WT lines; Odd_ratio: Odd_ratio from Fisher’s exact test (one-sided for enrichment); Mut_Dep_Lines: how many cell lines with the corresponding gene mutated and having CRISPR effect score <(-1.0); Mut_NotDep_Lines: how many lines with the corresponding gene mutated and but not having CRISPR effect score <(-1.0); NotMut_Dep_Lines: how many lines without the corresponding gene mutated but having CRISPR effect score <(-1.0); TotalLines: total number of qualified DepMap CCLE cell lines in the corresponding tissue type with both mutation status and CRISPR effect score data; DriverGenes: whether the gene is a oncogenic driver gene based on references (see note behind). Note: Only genes in green background are at significance of Adjusted p.value <0.05. Total lines of each tissue/tumor type with both mutation and DepMap CRISPR effect score data available have been tested for every of total 16925 genes and showed here are only the top ranked list. Oncogenic driver gene annotations are collated from an ICGC PCAWG-based study^8^ and two TCGA based studies^7,9^. The more powerful Barnard test, an alternative to Fisher’s exact test, indicated a significant KRAS p-value of 0.03 in pancreas (data not shown).


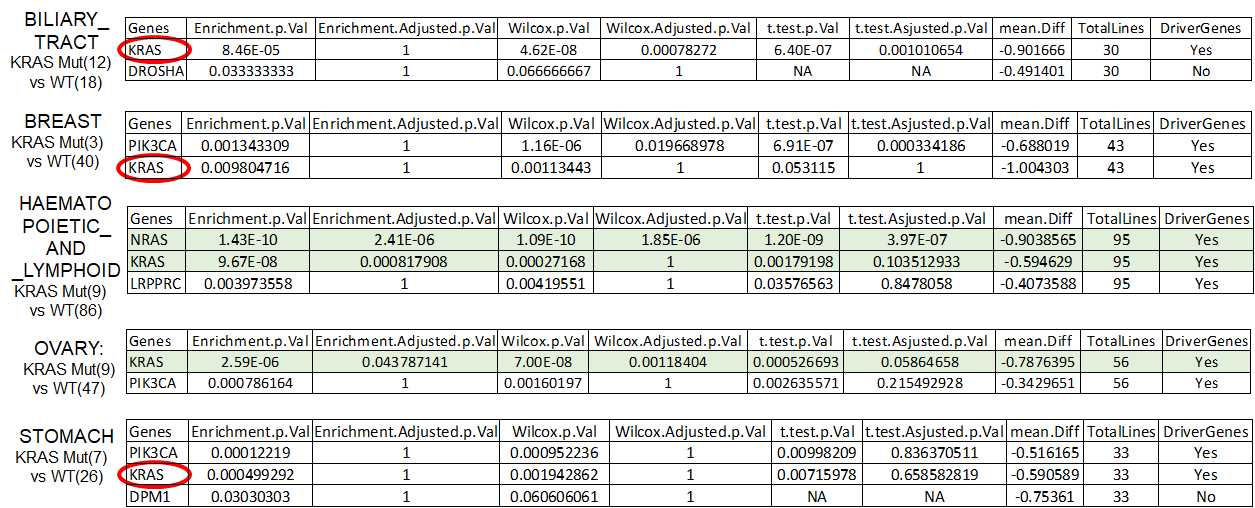


**Supplementary Figure 12.** KRAS or NRAS as the top gene with the most or nearly most significant association between presence of mutations in the corresponding gene and the dependency of this gene in many tissue types. Tables of the top genes out of ~17k genes with the most significant association between presence of mutations in a gene and the dependency on this corresponding gene denoted by its CRISPR effect score <(-1.0) (a score of -1 corresponds to the median of all common essential genes) across all cell lines from each of many tissue types. Significant association between the presence of mutations in the corresponding gene and the dependency of this gene within all the qualified DepMap CCLE cell lines were evaluated by Fisher's exact test with multiple-testing correction by Benjamini-Hochberg method and ranked by the enrichment p-values. Enrichment.p.Val: raw enrichment p-value was derived by Fisher’s exact test on a typical 2X2 contingency table created for all cell lines of each tissue type on status of a gene (whether the gene has mutation(s) or not versus whether it has a CRISPR effect scores <(-1.0) or not in each cell line); Enrichment.Adjusted.p.Val: adjusted Enrichment.p.Val by Benjamini-Hochberg method; Wilcox.p.val: p-value derived from Wilcoxon tests; Wilcox.Adjusted.p.Val: adjusted Wilcox.p.val by Benjamini-Hochberg method; t.test.p.val: p-value derived from t-test tests; t.test.Asjusted.p.Val: adjusted t.test.p.val by Benjamini-Hochberg method; mean.Diff: mean difference between the means of mutant lines vs WT lines; Odd_ratio: Odd_ratio from Fisher’s exact test (one-sided for enrichment); Mut_Dep_Lines: how many cell lines with the corresponding gene mutated and having CRISPR effect score <(-1.0); Mut_NotDep_Lines: how many lines with the corresponding gene mutated and but not having CRISPR effect score <(-1.0); NotMut_Dep_Lines: how many lines without the corresponding gene mutated but having CRISPR effect score <(-1.0); TotalLines: total number of qualified DepMap CCLE cell lines in the corresponding tissue type with both mutation status and CRISPR effect score data; DriverGenes: whether the gene is a oncogenic driver gene based on references (see note behind). Note: Only genes in green background are at significance of Adjusted p.value <0.05. Total lines of each tissue/tumor type with both mutation and DepMap CRISPR effect score data available have been tested for every of total 16925 genes and showed here are only the top ranked list. Oncogenic driver gene annotations are collated from an ICGC PCAWG-based study^8^ and two TCGA based studies^7,9^.


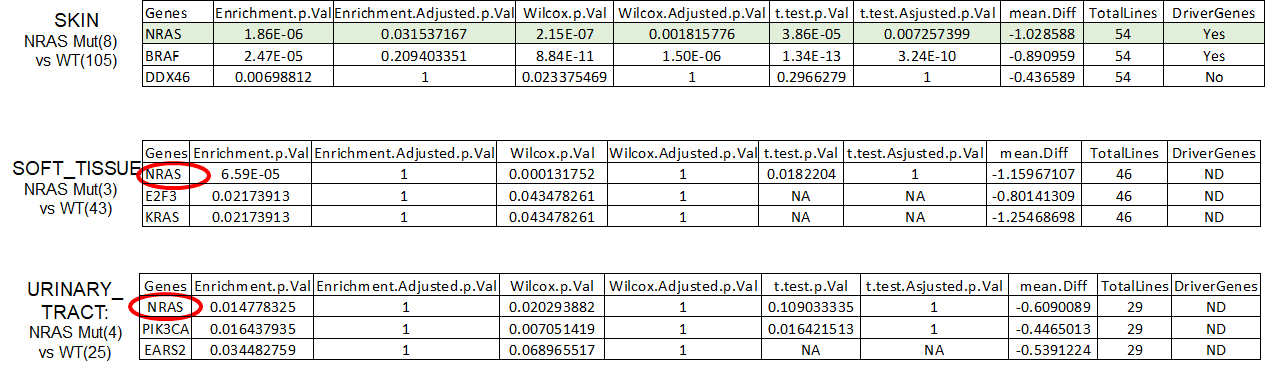


**Supplementary Figure 13.** NRAS as the top gene with the most or nearly most significant association between presence of mutations in the corresponding gene and the dependency of this gene in many tissue types. Tables of the top genes out of ~17k genes with the most significant association between presence of mutations in a gene and the dependency on this corresponding gene denoted by its CRISPR effect score <(-1.0) (a score of -1 corresponds to the median of all common essential genes) across all cell lines from each of many tissue types. Significant association between the presence of mutations in the corresponding gene and the dependency of this gene within all the qualified DepMap CCLE cell lines were evaluated by Fisher's exact test with multiple-testing correction by Benjamini-Hochberg method and ranked by the enrichment p-values. Enrichment.p.Val: raw enrichment p-value was derived by Fisher’s exact test on a typical 2X2 contingency table created for all cell lines of each tissue type on status of a gene (whether the gene has mutation(s) or not versus whether it has a CRISPR effect scores <(-1.0) or not in each cell line); Enrichment.Adjusted.p.Val: adjusted Enrichment.p.Val by Benjamini-Hochberg method; Wilcox.p.val: p-value derived from Wilcoxon tests; Wilcox.Adjusted.p.Val: adjusted Wilcox.p.val by Benjamini-Hochberg method; t.test.p.val: p-value derived from t-test tests; t.test.Asjusted.p.Val: adjusted t.test.p.val by Benjamini-Hochberg method; mean.Diff: mean difference between the means of mutant lines vs WT lines; Odd_ratio: Odd_ratio from Fisher’s exact test (one-sided for enrichment); Mut_Dep_Lines: how many cell lines with the corresponding gene mutated and having CRISPR effect score <(-1.0); Mut_NotDep_Lines: how many lines with the corresponding gene mutated and but not having CRISPR effect score <(-1.0); NotMut_Dep_Lines: how many lines without the corresponding gene mutated but having CRISPR effect score <(-1.0); TotalLines: total number of qualified DepMap CCLE cell lines in the corresponding tissue type with both mutation status and CRISPR effect score data; DriverGenes: whether the gene is a oncogenic driver gene based on references (see note behind). Note: Only genes in green background are at significance of Adjusted p.value <0.05. Total lines of each tissue/tumor type with both mutation and DepMap CRISPR effect score data available have been tested for every of total 16925 genes and showed here are only the top ranked list. Oncogenic driver gene annotations are collated from an ICGC PCAWG-based study^8^ and two TCGA based studies^7,9^.


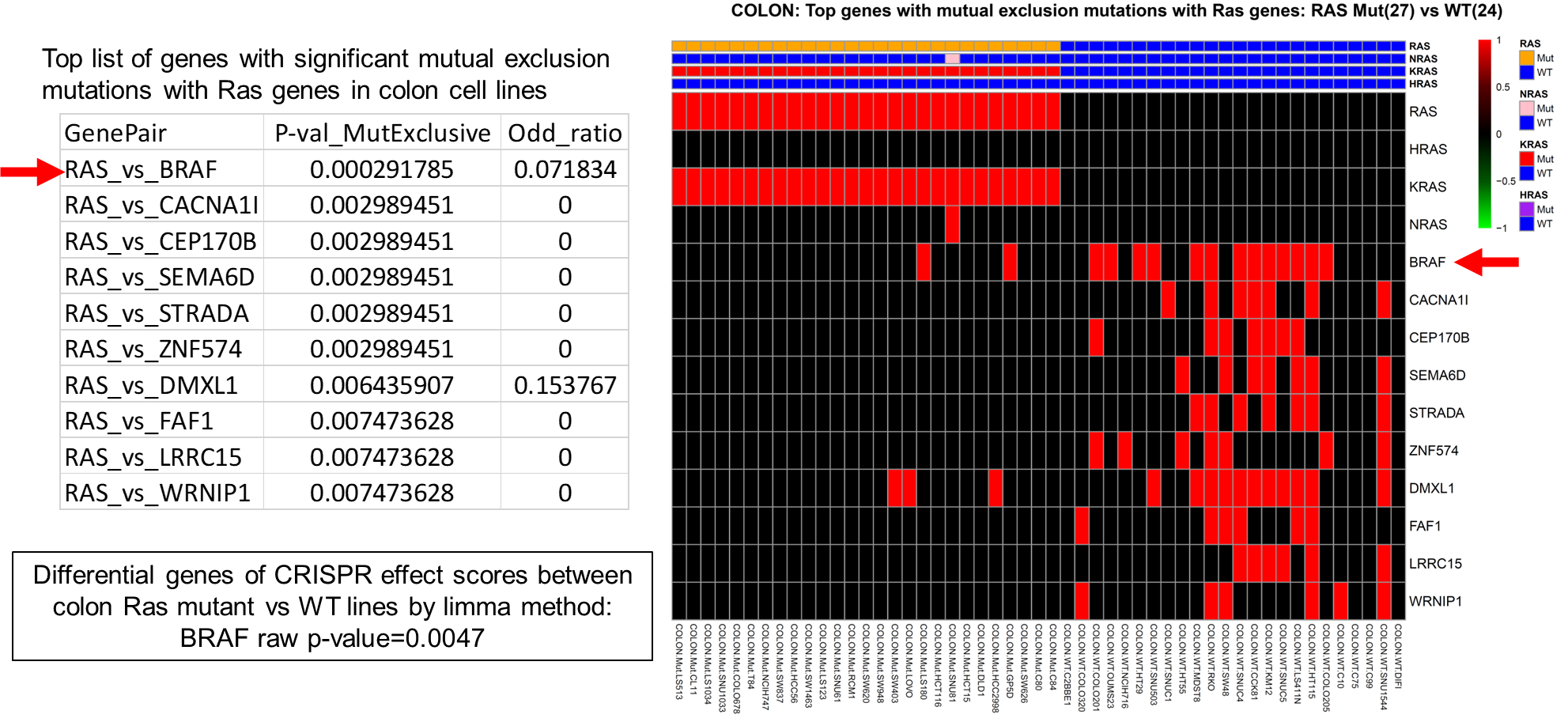


**Supplementary Figure 14.** BRAF was revealed likely as an essential gene for RAS WT lines with the most significant mutual exclusive mutations between RAS genes in colon cell lines. Left panel: Top list of genes with mutual exclusion mutations with RAS genes in colon cell lines. Right panel: heatmap of mutation status from Top list of genes with mutual exclusion mutations with RAS genes in colon cell lines. Mutual exclusion mutation assessment was done by Fisher’s exact test with 2X2 contingency table created for all cell lines (whether a cell line has a mutation of this corresponding gene or not versus whether it has RAS mutation(s) or not), which was used to assess the significance of mutual exclusion.

A.


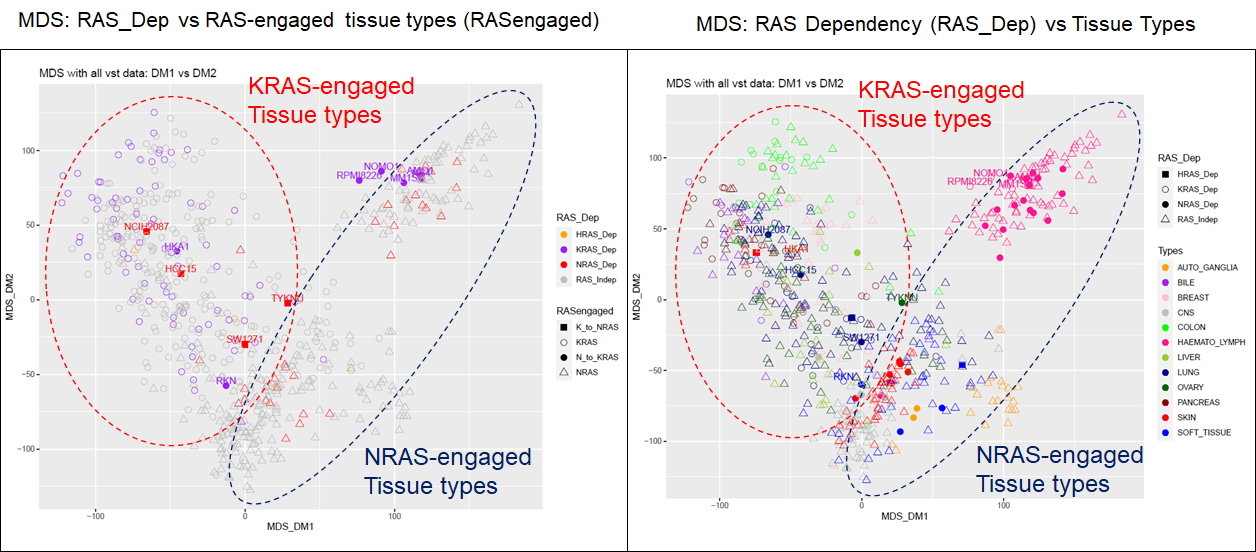


B.


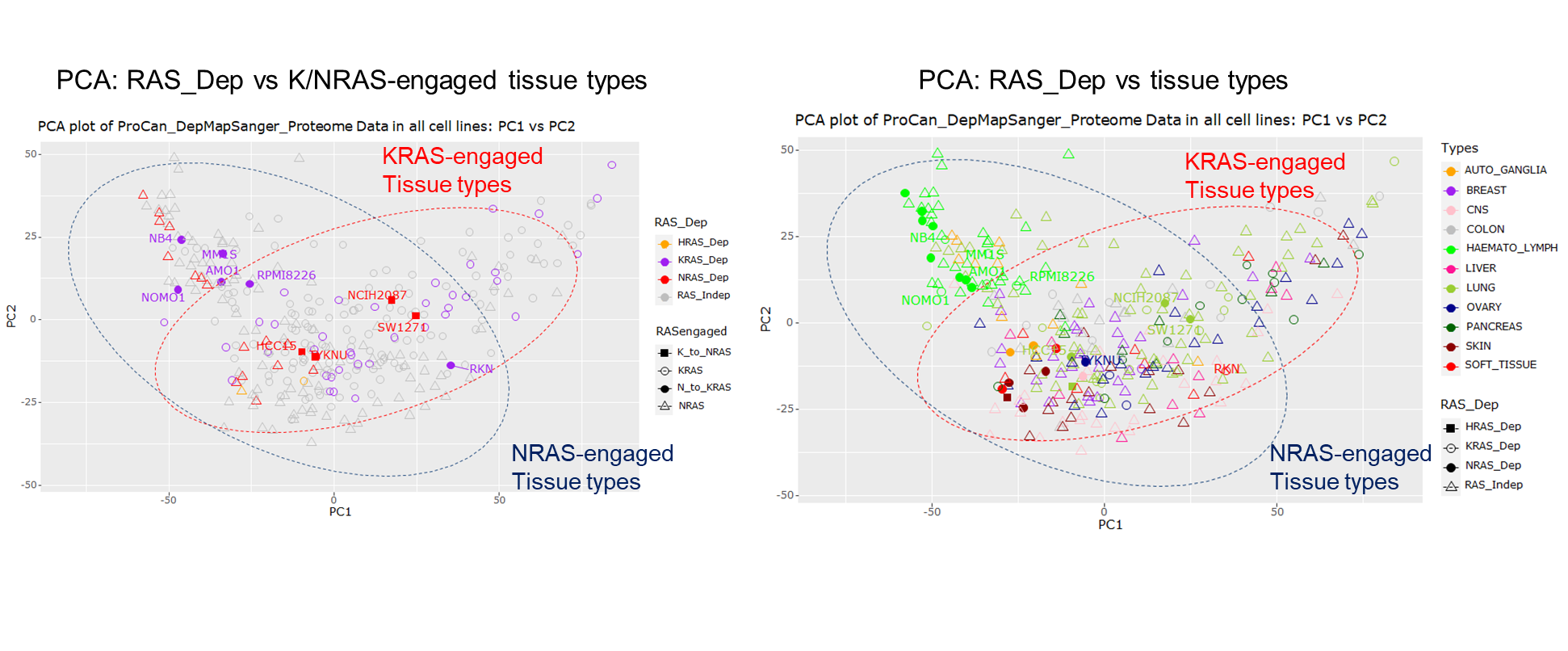


**Supplementary Figure 15.** MDS and PCA plots of RNAseq and proteome data revealed overall transcriptional and expression difference between cell lines from KRAS-engaged tissue types versus NRAS-engaged tissue types and the transcriptional/expression level impact caused by RAS mutations in RAS mutant lines comparing to the WT lines might be subtle if comparing to those differences between tissue types. **A.** MDS plots by RNAseq data of CCLE cell lines. **Left Panel.** RNAseq data revealed overall difference in transcriptional profiles between cell lines from KRAS-engaged tissue types highlighted in a large red circle versus NRAS-engaged tissue types highlighted in a large blue circle, which supported the idea of KRAS- or NRAS-engaged tissue types that inferred from the differential analysis of DepMap CRISPR effect data. Cell lines are categorized by the shapes of the data points for RAS-engaged tissue types (RASengaged): K_to_NRAS (filled rectangles), which are the 4 converted lines (also labeled by cell line names) described in **Supplementary Figure 8B**; KRAS (open circles): KRAS-engaged tissue types; N_to_KRAS(closed circles), which are the 7 converted lines (also labeled by cell line names) described in **Supplementary Figure 8C**; NRAS(open triangles): NRAS-engaged tissue types; Cell lines are also colored by their RAS-Dependency types (RAS_Dep), which are tentatively defined by each observed threshold of RAS genes (K-, N-,H-) for distribution of CRISPR effect scores revealed by **Supplementary Figure 8A, 8D:** KRAS_Dep (KRAS dependent, with negative CRISPR effect scores <(-1); NRAS_Dep (NRAS dependent, with negative CRISPR effect scores <(-0.75); HRAS_Dep (HRAS dependent, with negative CRISPR effect scores <(-0.75). (the slight variation of the thresholds for denoting the RAS_Dep types does not change the overall observations). **Right Panel.** The same MDS plot except with cell lines colored by their actual tissue types and shaped for their RAS_Dep (RAS Dependency status as defined in **Left Panel**). **B.** PCA plots by proteome data of CCLE cell lines. **Left Panel.** Proteome data revealed overall difference in expression profiles between cell lines from KRAS-engaged tissue types highlighted in a large red circle versus NRAS-engaged tissue types highlighted in a large blue circle, which supported the idea of KRAS- or NRAS-engaged tissue types that inferred from the differential analysis of DepMap CRISPR effect data. Cell lines are categorized by the shapes of the data points for their actual tissue types; **Right Panel.** The same PCA plot except with cell lines colored by their actual tissue types and shaped for their RAS_Dep (RAS Dependency status as defined in **Left Panel**). Both RNAseq and proteome data suggested that the impact of RAS mutations on the difference between (K- or N-) RAS mutant vs WT lines could be subtle on top of the tissue-specific expression profiles, or such impact would confer through other mechanisms such as signaling or post-transcriptional modifications etc that may be explored using other omics data. Cell lines are also in different shapes for their RAS-Dependency types (RAS_Dep), which are tentatively defined similar to **Left Panel**. All converted lines are labeled by their cell line names. MDS: multi-dimensional scaling.


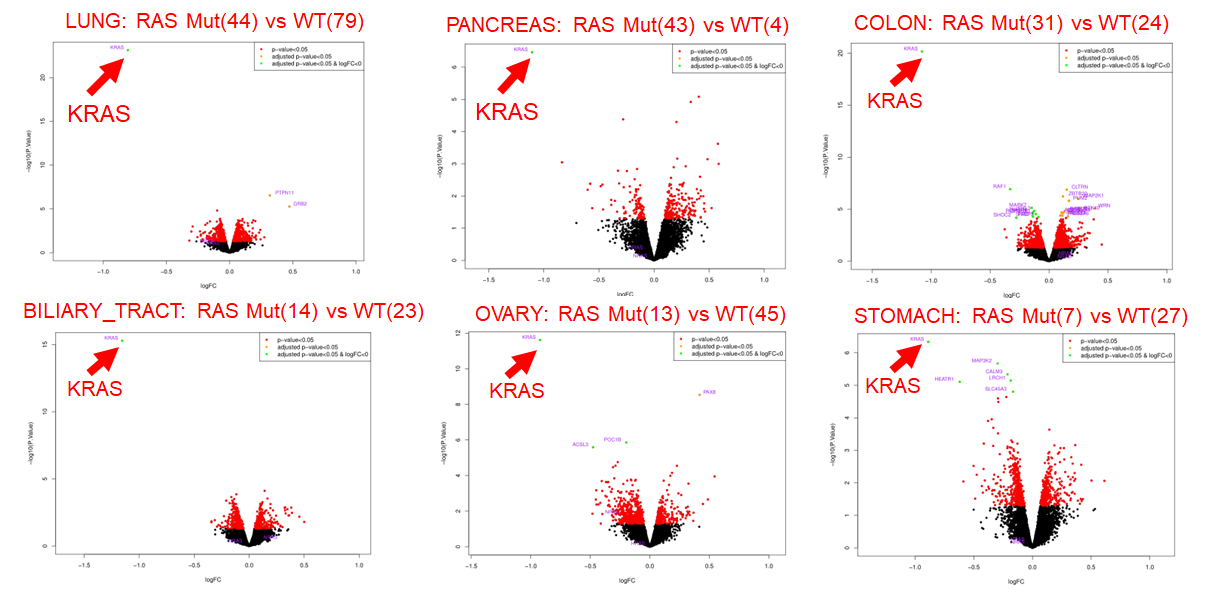


**Supplementary Figure 16.** Differential gene analysis of DepMap CRISPR gene effect data between RAS mutant vs WT lines in a subset of tissue types using DepMap version 23Q2. Comparison of DepMap version 23Q2 vs version 21Q1: both versions consistently showed that KRAS is the most or nearly most significantly differential gene for CRISPR gene effect at adjusted p-value<0.05 with the most negative dependency difference between RAS mutant vs WT lines from a set of tissue types. KRAS is the most significantly differential gene for CRISPR gene effect at adjusted p-value<0.05 with the most negative dependency difference between RAS mutant vs WT lines from many tissue types. Volcano plots of all genes for CRISPR effect score data of DepMap between RAS mutant vs WT lines from many different tissue origins including colon, breast, ovary, biliary tract. Green data points: genes with significant adjusted p-value (<0.05) for multiple testing and logFC<0; orange data points: genes with significant adjusted p-value (<0.05) for multiple testing and logFC>=0; red data points: genes with significant nominal p-value (<0.05); black data points: genes without statistical significance. The parentheses after “Mut” or “WT” indicate number of mutant lines or number of WT lines, respectively. Note: limma model is set up on the whole dataset including all tumor types, and so all data is under the same roof of the limma model, by which the power of the analysis was essentially increased as described earlier (Ritchie et al 2015, *Nucleic Acids Res*., 43(7):e47). X-axis logFC: log2 fold change as for the actual difference of the CRISPR effect scores between RAS mutant vs WT lines in volcano plots, since the values of CRISPR effect scores inherently in logarithm transformed scale were used directly in limma; y-axis –log10(p.Value): (-1)*log10 of raw p-value of limma analysis.

Colon: LARGE_INTESTINE.


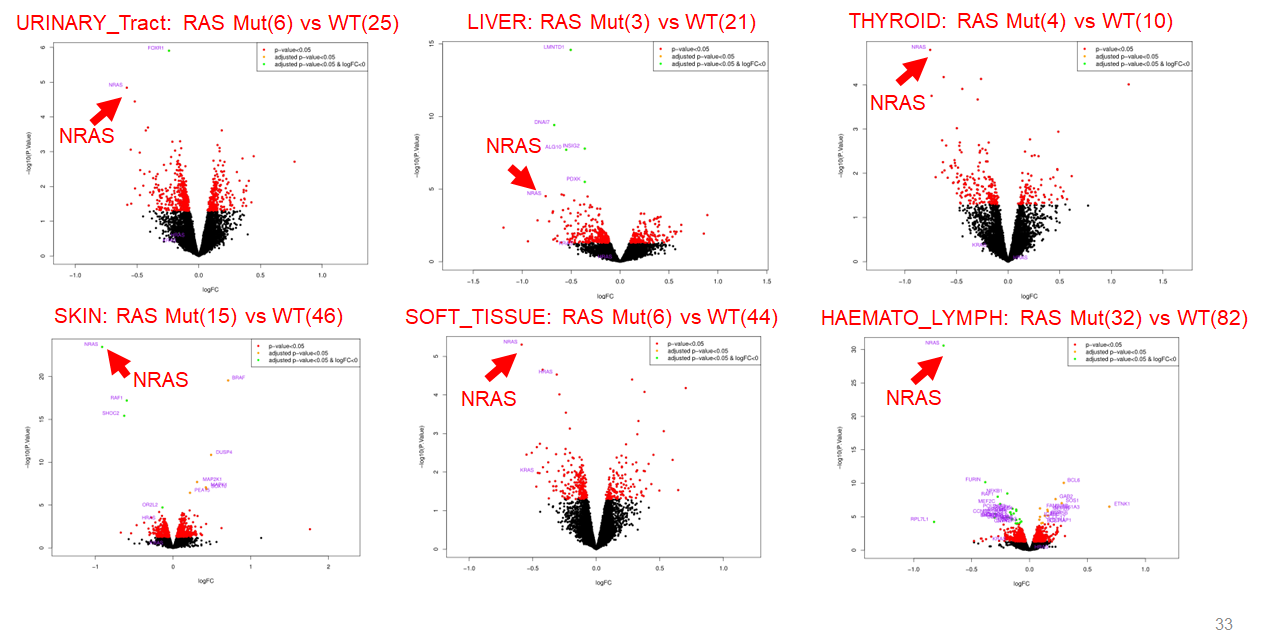


**Supplementary Figure 17.** Differential gene analysis of DepMap CRISPR gene effect data between RAS mutant vs WT lines in another subset of tissue types using DepMap version 23Q2. Comparison of DepMap version 23Q2 vs version 21Q1: both versions consistently showed that NRAS is the most significantly differential gene for CRISPR gene effect at adjusted p-value<0.05 (with exception of SOFT_TISSUE at raw p-value<0.05) with the most negative dependency difference between RAS mutant vs WT lines from another set of tissue types. Volcano plots of all genes for CRISPR effect score data of DepMap between RAS mutant vs WT lines from many different tissue origins. Green data points: genes with significant adjusted p-value (<0.05) for multiple testing and logFC<0; orange data points: genes with significant adjusted p-value (<0.05) for multiple testing and logFC>=0; red data points: genes with significant nominal p-value (<0.05); black data points: genes without statistical significance. The parentheses after “Mut” or “WT” indicate number of mutant lines or number of WT lines, respectively. Note: limma model is set up on the whole dataset including all tumor types, and so all data is under the same roof of the limma model, by which the power of the analysis was essentially increased as described earlier (Ritchie et al 2015, *Nucleic Acids Res*., 43(7):e47). X-axis logFC: log2 fold change as for the actual difference of the CRISPR effect scores between RAS mutant vs WT lines in volcano plots, since the values of CRISPR effect scores inherently in logarithm transformed scale were used directly in limma; y-axis –log10(p.Value): (-1)*log10 of raw p-value of limma analysis.

Colon: LARGE_INTESTINE.


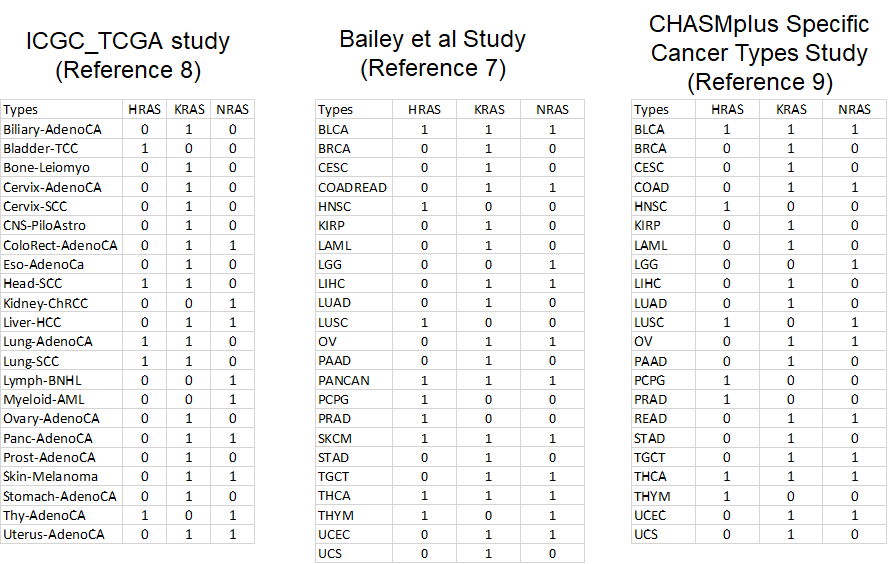


**Supplementary Figure 18.** RAS genes were assigned as driver genes of oncogenesis in a wide range of tissue/tumor types revealed by computational studies^7, 8, 9^ using genomic data. Numbers in tables, 1: rated as oncogenic driver genes; 0: rated not as oncogenic driver genes.
